# Automated Approaches of Text Simplification of Patient Education Materials: Scoping Review

**DOI:** 10.2196/88365

**Published:** 2026-05-07

**Authors:** Cornelia Krenn, Christine Loder, Natalie Berger, Klaus Jeitler, Thomas Semlitsch, Andrea Siebenhofer, Denise Wilfling

**Affiliations:** 1 Institute of General Practice and Evidence-based Health Services Research, Medical University of Graz Graz Austria; 2 Institute for Medical Informatics, Statistics and Documentation, Medical University of Graz Graz Austria; 3 Institute of General Practice, Goethe University Frankfurt am Main Frankfurt Germany; 4 Institute of Nursing and Health Sciences, Medical University of Graz Graz Austria

**Keywords:** artificial intelligence, large language models, patient education materials, automatic text simplification, linguistic quality, content fidelity

## Abstract

**Background:**

Patient education materials (PEMs) often exceed the American Medical Association’s (AMA) recommended sixth-grade reading grade level (RGL). While artificial intelligence (AI) offers potential for automated text simplification, concerns persist regarding linguistic quality, content fidelity, and the understandability of simplified PEMs by laypeople.

**Objective:**

This scoping review maps existing evidence on automated language processing technologies for simplifying PEMs for laypeople.

**Methods:**

Following the Joanna Briggs Institute (JBI) methodology and the PRISMA-ScR (Preferred Reporting Items for Systematic Reviews and Meta-Analyses extension for Scoping Reviews) guideline, 5 bibliographic databases (Ovid MEDLINE, Embase, CINAHL, PsycInfo, and IEEE Xplore) were systematically searched from 2019 to May 2025, supplemented by reference screening and gray literature searches. Eligible sources were peer-reviewed empirical studies published in English that examined large language models (LLMs), AI-supported writing assistants, AI-based conversational agents, or AI-supported tools designed for automatic text simplification of PEMs. Targeted outcomes included linguistic quality (ie, linguistic comprehensibility, linguistic correctness) and content fidelity (ie, factual accuracy, factual completeness) of simplified PEMs. Excluded sources comprised rule-based systems, manual text simplification, non-laypeople target groups, and technology-focused performance metrics. Results were synthesized via thematic analysis across the domains of targeted outcomes. In accordance with JBI methodology, a risk-of-bias assessment was not performed.

**Results:**

A total of 31 eligible studies met the inclusion criteria, examining various LLMs, including OpenAI’s GPT series, Gemini, Bard, Claude, Copilot, and Llama. Specifically, GPT-4.0 achieved the most consistent improvements in standardized readability metrics (eg, the Flesch-Kincaid Grade Level [FKGL]). However, achieving predefined target RGLs remained challenging across all LLMs, particularly at lower RGLs. Findings on content fidelity were inconsistent: despite high content similarity scores, content accuracy was often compromised.

**Conclusions:**

This is the first scoping review to comprehensively synthesize evidence on automated technologies for text simplification in PEMs. The review identified 2 critical validation gaps. First, no study examined the linguistic correctness (eg, grammar and typographical errors) of automatically simplified PEMs. Second, and most notably, the understandability of the simplified PEMs was assessed exclusively by experts, with no empirical evaluation involving laypeople. Although LLMs effectively reduce text complexity as measured by objective readability metrics, reliance on these formulas represents a critical limitation, as they serve merely as structural proxies. Improvements in readability do not guarantee the maintenance of content accuracy or laypeople’s understandability. Current evidence is further limited by the lack of systematic prompt quality evaluation and the predominant focus on English-language PEMs in US contexts, restricting generalizability. This review provides a foundation for future research by highlighting the need for validated evaluation frameworks that encompass layperson testing and content verification. For clinical practice, LLMs should currently serve as assistive tools, with mandatory expert review remaining essential to verify content fidelity before disseminating LLM-simplified PEMs to laypeople.

## Introduction

### Rationale

Effective health communication is recognized as a public health priority [1]. Health communication aims to improve health by ensuring effective understanding and application of health information. Central to this is health literacy, defined as the degree to which individuals have the capacity to obtain and understand health information needed to make appropriate health decisions [2]. Patients with higher health literacy are more likely to engage in health-promoting behaviors, utilize health care services, and effectively manage chronic diseases [3]. However, the use of complex medical language poses a significant barrier to patient understanding. Health care professionals are encouraged to use plain language tailored to patients’ comprehension levels, which can be challenging, especially in time-sensitive clinical care settings [4,5]. Patient education materials (PEMs) play a central role in supporting patient-physician interactions by providing clear and accessible information on health conditions, treatments, and health promotion [6]. Personalized PEMs have been shown to improve patient care through shared decision-making, enhanced patient satisfaction, and better physical and psychosocial well-being [7]. To maximize accessibility of PEMs, leading organizations such as the National Institutes of Health and the American Medical Association (AMA) recommend writing PEMs at or below a sixth-grade reading level (RGL) [8,9]. However, numerous studies have demonstrated that a significant portion of existing PEMs fail to meet this benchmark, often being written at a level too complex for many patients to understand. An analysis of PEMs showed average readability scores ranging from 8th to 15th RGL across various medical fields [10-16]. This readability concern has not improved between 2001 and 2022. These findings underscore that simplified versions of PEMs are necessary, and health care professionals are encouraged to provide easy-to-read PEMs to patients [17,18].

Artificial intelligence (AI) offers promising opportunities to enhance effective health communication. In particular, large language models (LLMs) have emerged as transformative tools in natural language processing (NLP), enabling diverse applications such as answering patient questions; summarizing, translating, or simplifying medical texts; supporting clinical paperwork; and providing individualized medical guidance [19-21]. Importantly, LLMs have the potential to enhance the accessibility of medical knowledge by making complex medical language more comprehensible to laypeople, thereby enabling patients to better understand their health conditions [22,23]. Text simplification as an NLP task has advanced significantly in recent years, especially driven by developments in LLMs since 2019. Whereas earlier technologies relied primarily on rule-based systems or machine learning models, LLMs have enabled a paradigm shift in NLP, making these capabilities more widely accessible to health care professionals and health researchers. In addition, LLM capabilities have rapidly evolved, demonstrating increasingly sophisticated language understanding and generation abilities [24,25].

Despite promising results, important challenges remain. These include the risk of factual errors (hallucinations), critical omissions of information, and the unintended loss of meaning between original and simplified text versions. Furthermore—and most critically—the difficulty of verifying understandability persists, as strong performance on standard quality metrics does not guarantee that simplified texts are actually understandable to laypeople [26]. Moreover, the rapid evolution of AI language processing technologies makes it challenging for researchers and health care providers to maintain an up-to-date overview of available tools and supporting evidence.

### Objectives

This scoping review aimed to identify and map existing evidence on the use of automated language processing technologies for the simplification of PEMs into layperson-friendly language. For this review, layperson-friendly language is defined as text characterized by an accessible reading level, simple sentence structures, and explanation or avoidance of medical jargon. This scoping review is guided by the following research questions:

- What automated language processing technologies are currently used to simplify PEMs into layperson-friendly language?
- How are the linguistic quality and content fidelity of these simplified texts evaluated?

## Methods

### Review Principles and Protocol

This scoping review followed the methodological framework of the Joanna Briggs Institute (JBI) [27] and is reported according to the PRISMA-ScR (Preferred Reporting Items for Systematic Reviews and Meta-Analyses extension for Scoping Reviews) [28] (Multimedia Appendix 1) and the PRISMA-S (PRISMA Statement for Reporting Literature Searches in Systematic Reviews) [29] (Multimedia Appendix 2) guidelines. The review methodology was registered in the Open Science Framework [30].

### Deviation From the Protocol

**Figure 1:**
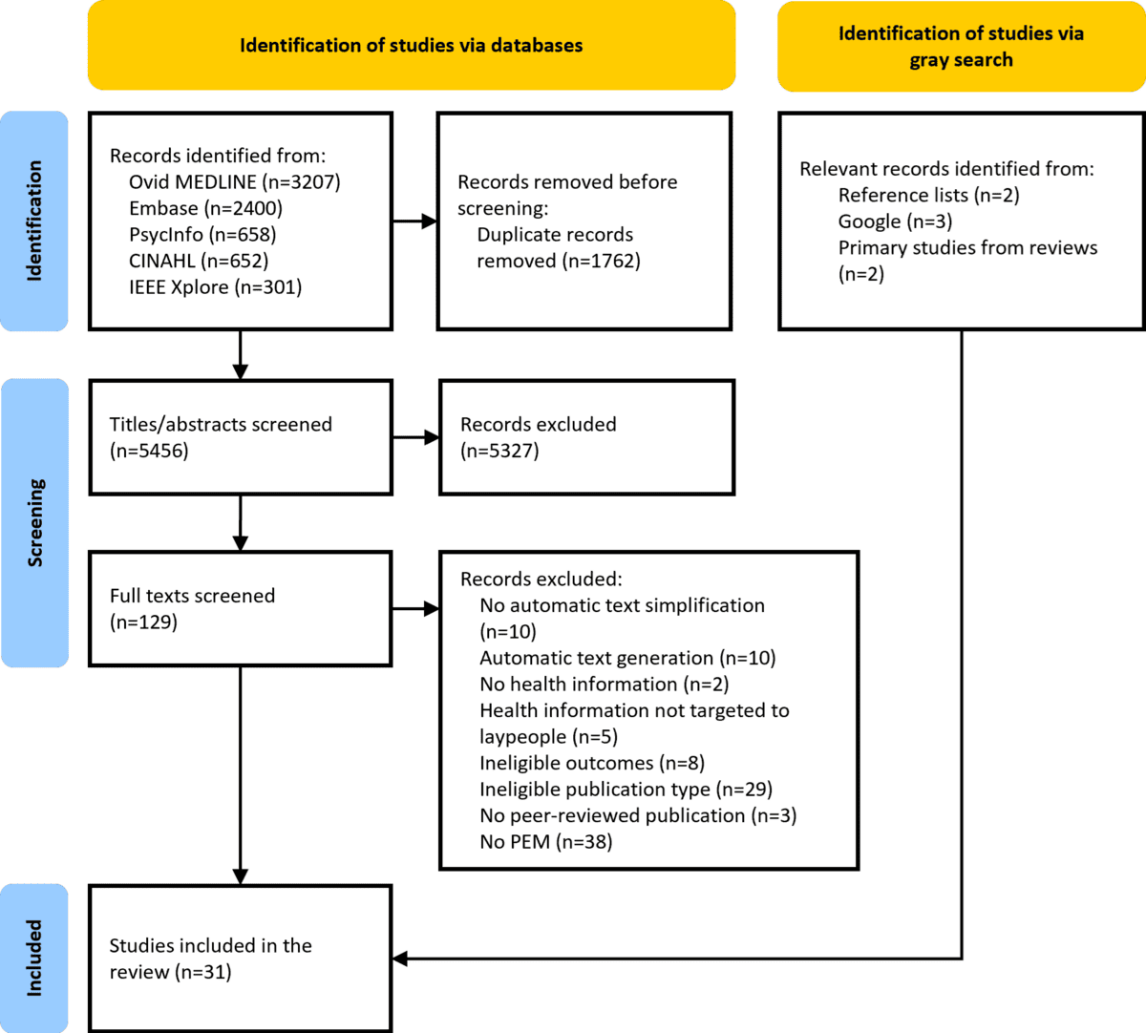
PRISMA (Preferred Reporting Items for Systematic Reviews and Meta-Analyses) flow diagram for study selection. PEM: patient education material.

The preregistered protocol specified the inclusion of studies on automatic text simplification of any complex medical or health-related text. As Figure 1 shows, full-text screening revealed a large and highly heterogeneous body of literature, encompassing different document types such as radiology reports, electronic health records, discharge letters, PEMs, informed consent forms, and scientific papers. This heterogeneity presents challenges for meaningful synthesis and comparison across fundamentally different health information materials with varying purposes, audiences, and complexity levels.

Additionally, this study was framed as part of a larger project, the A+CHIS project [31], which aims to develop a system that provides users with a selection of diverse health documents and preliminary PEMs tailored to their individual information needs and cognitive prerequisites. To present a more focused and coherent synthesis of evidence that would yield insights for patient-facing health communication systems such as A+CHIS, a post hoc decision was made to refine the scope to studies concerning PEM simplification specifically. This narrowed focus aligns with the primary goal of improving health information accessibility for laypeople and ensures methodological consistency in evaluating simplification approaches.

This post hoc decision was made after the initial full-text screening but before full-text data extraction. All other methods remained unchanged from the preregistered protocol.

### Screening and Identifying Relevant Studies: Information Sources and Search

A comprehensive literature search was conducted in May 2025 across 5 bibliographic databases: MEDLINE, Embase, CINAHL, PsycINFO, and IEEE Xplore. The MEDLINE and Embase strategies were run simultaneously as a multifile search in Ovid, and results were deduplicated using the Ovid deduplication tool. The search strategy was based on database-specific controlled vocabulary and free-text terms. To identify relevant search terms, the MeSH Browser, a keyword analysis tool [32], and a synonym identification tool [33] were used. The search was limited to the period from 2019 to 2025, as this period marks significant advances in automated language processing technologies, particularly with the emergence of LLMs that have substantially enhanced automatic text simplification capabilities. No search filter was applied. The search block for “automated language processing technologies” was based on 2 existing search strategies [34,35]. Detailed search strategies for the databases are provided in Multimedia Appendix 3. The MEDLINE search strategy was peer reviewed by another research team member (KJ).

Reference lists of included studies were manually screened to identify additional studies. We also searched gray literature in July 2025 using the search string “patient education material” AND (“large language model” OR “ChatGPT”) AND “simplifying” in Google. The first 5 pages of each search result were screened. We did not contact the authors of included studies for clarification, as the available data were sufficient to conduct the scoping review. No other methods were used to locate additional studies. Eligible studies were imported into EndNote 21.5 (Clarivate Plc) to identify and remove duplicates.

### Eligibility Criteria

#### Framework Applied

The Population-Concept-Context (PCC) framework was used to define the eligibility criteria and guide the search strategy [27].

- Population:
  The target population was defined as laypeople requiring PEMs, including patients and their families, the general public, individuals with limited health literacy, and health care professionals without specific medical expertise.
- Concept:
  The central concept was the use of automated language processing technologies for the simplification of PEMs.
- Context:
  The context was limited to PEM simplification, defined as any text (full text or excerpt) designed to inform laypeople about medical or health-related topics. Nontext formats, such as video, audio materials, or infographics, were not considered in this review.

Building upon the PCC framework, the following inclusion and exclusion criteria were applied for study selection:

#### Inclusion Criteria

- Studies that assessed automated language processing technologies, specifically LLMs such as AI-supported writing assistants (eg, DeepL Write) and AI-based conversational agents (eg, ChatGPT, Gemini), or other AI-supported tools designed for automatic text simplification.
- Studies that assessed text quality indicators of simplified PEMs in at least one of two domains: (1) linguistic quality, encompassing linguistic comprehensibility (eg, readability, text complexity, word choice, structural clarity, medical jargon) and linguistic correctness (eg, grammatical accuracy, typographical errors); and (2) content fidelity, encompassing factual correctness (eg, hallucinations, exaggerations, falsifications, understatements, misinterpretations) and factual completeness (eg, content retention).
- Studies that measured outcomes either objectively (eg, FKGL) or subjectively (eg, expert ratings, user feedback).
- Peer-reviewed empirical study designs (quantitative, qualitative, and case studies) published in English, including primary studies identified through relevant reviews.

#### Exclusion Criteria

- Studies that evaluated nonlearning or rule-based systems, manual text simplification or human postediting of AI output, automated language translation without simplification intent, or text analysis software tools.
- Studies that evaluated the effectiveness of automated simplified PEMs targeted at health care professionals.
- Studies that evaluated only technology-oriented performance metrics (eg, Bilingual Evaluation Understudy [BLEU] or Recall-Oriented Understudy for Gisting Evaluation [ROUGE] scores).
- Non–peer-reviewed publications (eg, preprints, editorials, commentaries, letters to the editor, opinions), studies lacking a complete methodological description, and publications in languages other than English.

### Selection of Sources of Evidence

Following JBI guidelines [27], 2 authors (CK, DW, CL, or NB) independently screened all titles and abstracts against the inclusion criteria. A pilot stage involving 25 titles and abstracts was conducted to ensure consistent application of the criteria, with conflicts resolved through discussion. Full texts of potentially eligible studies were subsequently assessed by 2 independent reviewers (CK, DW, CL, or NB) using the same eligibility criteria. Disagreements were resolved through discussion or by involving a third reviewer (CK, DW, CL, or NB).

### Data Charting

A standardized data extraction form was developed and piloted on 3 eligible studies to assess its clarity and completeness. Following the pilot test and discussion within the research team, 1 additional item (the language of simplified PEMs) was added to the final form.

### Data Items

The following data from the included studies were extracted: bibliographic details (authors, year of publication, and country), technology details (name and version of LLM and prompts used), source text (medical field of PEMs, number of analyzed materials, and language), outcomes (text quality indicators and measurement methods), and key findings (main results related to linguistic quality and content fidelity). As recommended by the JBI methodology for scoping reviews [27], 1 reviewer (CK) extracted the data, which was verified by a second reviewer (DW).

### Critical Appraisal

In line with JBI guidelines [27], critical appraisal of eligible studies was not required.

### Collating, Summarizing, and Reporting the Results

Following the JBI scoping review methodology [27], we conducted a descriptive synthesis of the extracted data. We did not perform an analytical synthesis of outcomes but instead mapped and summarized findings descriptively. The extracted data were collated iteratively and organized using a framework-based approach, categorizing findings into the 2 predefined outcome domains: linguistic quality (encompassing linguistic comprehensibility and linguistic correctness) and content fidelity (encompassing factual correctness and factual completeness). Within each domain, descriptive qualitative content analysis was conducted by the first author (CK) to summarize the types of automated language processing technologies evaluated, the measurement methods applied, and the reported direction of effect (eg, improvements in readability scores or identified factual inaccuracies). Results were presented descriptively through narrative summaries, tables, and figures to address the review questions. Frequency counts were used where appropriate to quantify the occurrence of specific technologies or outcome measures. All descriptive analyses were conducted using Microsoft Excel.

## Results

### Selection of Sources of Evidence

The systematic database search identified 7218 references. Following deduplication, 5456 titles and abstracts were screened, and 129 full texts were subsequently assessed for eligibility. This process resulted in the inclusion of 24 [36-59] studies specifically addressing PEMs. In addition, 2 studies [60,61] identified from relevant reviews were included. Two further studies [62,63] were identified through reference list screening of the included studies, and a supplementary Google search yielded 3 additional relevant studies [64-66]. In total, this scoping review included 31 studies focusing on PEMs. Figure 1 shows the study selection process in a PRISMA (Preferred Reporting Items for Systematic Reviews and Meta-Analyses) flow diagram.

Studies excluded at the full-text screening stage and their primary reasons for exclusion are provided in Multimedia Appendix 4.

### Characteristics of Sources of Evidence

The main characteristics of all included studies are presented in Table 1. All included studies investigated LLMs exclusively, evaluating 8 different models. OpenAI’s GPT series emerged as the most extensively studied models: GPT-4.0 (n=10) [36-43,62,64], GPT-3.5 (n=8) [44-49,60,61], and GPT-3.0 (n=1) [50]. Other evaluated LLMs included Google’s Gemini (n=4) [51,52,65,66] and Bard (n=5) [53-56,63], Anthropic’s Claude (n=2) [57,65], Microsoft’s Copilot (n=1) [66], and Meta’s Llama (n=1) [57], with 12 studies conducting comparative analyses across multiple models [51-59,63,65,66]. As illustrated in Multimedia Appendix 5, all included studies were published very recently, with 11 in 2025 [36-40,44,51,57,64-66], 19 in 2024 [41-43,45-48,50,52-56,58-63], and 1 in 2023 [49]. Although the literature search covered the period from 2019 onward, no relevant studies were identified before 2023, highlighting the emerging nature of this field.

**Table 1:** Summary of main characteristics of the included studies.

Study	Country	Study design	LLM^a^; number of PEMs^b^ analyzed (language of PEMs)	Medical field in PEMs	Readability scale	Target RGL^c,d^
Spina et al [36]	United States	Not reported	GPT-4.0; 9 (English)	Glaucoma	FKGL^e^ and FRE^f^	5
Reaver et al [37]	United States	Cross-sectional study	GPT-4.0; 57 (English) and 56 (Spanish)	Orthopedic surgery	FRE, Fry, LIX^g^, RIX^h^, SMOG^i^, GPM^j^ Fry, FHRI^k^, and SOL^l^	6
Picton et al [38]	United States	Not reported	GPT-4.0; 340^m^ (English)	Neurology and neurological surgery	FKGL and FRE	5
Li et al [44]	United States	Cross-sectional comparative study	GPT-3.5; 50 (English)	Cataract surgery	FKGL, SMOG, GFI^n^, and CLI^o^	5
Dihan et al [51]	United States	Cross-sectional comparative study	GPT-3.5, GPT-4.0, and Gemini Advanced; 20 (English)	Dry eye disease	FKGL and SMOG	6
Chandra et al [39]	United States	Not reported	GPT-4.0; 30 (English)	Orthopedic surgery	SMOG	6-8
Busigo Torres et al [40]	United States	Not reported	GPT-4.0; 77 (Spanish)	Orthopedic	FHRI and SOL	5
Andalib et al [57]	United States	Not reported	GPT-3.5, GPT-4.0, Claude 2, and Llama 2; 48 (English)	Orthopedic	FKGL and FRE	5
Will et al [65]	United States	Cross-sectional study	GPT-4.0, Gemini 1.5-flash, and Claude 3.5 Sonnet; 60 (English)	Heart disease, cancer, and stroke	FKGL, FRE, SMOG, and GFI	5
Naghdi et al [66]	The Netherlands	Comparative observational study	GPT-3.5, GPT-4.0, Copilot, and Gemini; 30 (English)	Reproductive genetics	FKGL, FRE, SMOG, GFI, CLI, and LWF^p^	6-8
Singh et al [64]	United States	Not reported	GPT-4.0; 25 (English)	Neurosurgery	FKGL, FRE, SMOG, CLI, and ARI^q^	8
Zaki et al [41]	United States	Not reported	GPT-4.0; 73 (English)	Interventional radiology procedures	FRE, GFI, and ARI	5
Vallurupalli et al [45]	United States	Not reported	GPT-3.5; 18^r^ (English)	Hand surgery	Combined calculator: FKGL, FRE, SMOG, ARI, GFI, LWF, and CLI	6-8
Shehab et al [42]	United States	Cross-sectional study	GPT-4.0; 124 (English)	Cleft lip and palate	FKGL and FRE	6
Patel et al [43]	United States	Proof‐of‐concept study	GPT-4.0; 71 (English)	Otolaryngology	FKGL, FRE, SMOG, and GFI	6
Oliva et al [46]	United States	Cross-sectional study	GPT-3.5; 109 (English)	Otolaryngology	FKGL and FRE	5
Kianian et al [53]	United States	Not reported	GPT-4.0 and Bard; 9 (English)	Uveitis	FKGL	6
Rasika et al [47]	United States	Not reported	GPT-3.5; 15 (English and Spanish)	Ophthalmology	FKGL, FRE, GFI, FHRI, Crawford Nivel-de-Grado, Gutiérrez, Szigriszt-Pazos/INFLESZ^s^, and Legibilidad-μ	Nr
Gupta et al [52]	United States	Not reported	GPT-4.0 and Gemini; 7 (English)	Radiology	FKGL, FRE, SMOG, and GFI	6
Garcia Valencia et al [58]	United States and Thailand	Not reported	GPT-3.5 and GPT-4.0; 27^t^ (English)	Living kidney donation	FKGL	8
Fanning et al [59]	United States	Not reported	GPT-3.5 and GPT-4.0; 75 (English)	Plastic surgery	FKGL, FRE, Fry, SMOG, GFI, and Raygor Estimate	6
Dihan et al [54]	United States	Cross-sectional comparative study	GPT-3.5, GPT-4.0, and Bard; 20 (English)	Idiopathic intracranial hypertension	FKGL and SMOG	6
Dihan et al [55]	United States	Cross-sectional comparative study	GPT-3.5, GPT-4.0, and Bard; 20 (English)	Childhood glaucoma	FKGL and SMOG	6
Dihan et al [56]	United States	Cross-sectional comparative study	GPT-3.5, GPT-4.0, and Bard; 20 (English)	Pediatric cataract	FKGL and SMOG	6
Baldwin [50]	United Kingdom	Not reported	GPT-3.0; 50 (English)	Burns first aid	FKGL, FRE, SMOG, GFI, and CLI	6
Ayre et al [48]	Australia	Observational study	GPT-3.5; 26^u^ (English)	Not restricted^v^	SMOG	8
Manasyan et al [60]	United States	Not reported	GPT-3.5; 34 (English)	Alveolar bone grafting	FKGL, FRE, and GFI	5
Vallurupalli et al [61]	United States	Not reported	GPT-3.5; 18 (English)	Craniofacial procedures	Combined calculator: FKGL, FRE, SMOG, ARI, GFI, LWF, and CLI	8
Abreu et al [62]	United States	Cross-sectional study	GPT-4.0; 34 (English)	Cancer	FKGL, SMOG, Fry, and GFI	6
Rouhi et al [63]	United States	Pilot study	GPT-3.5 and Bard; 21 (English)	Aortic stenosis	FKGL, FRE, SMOG, and GFI	5
Kirchner et al [49]	United States	Proof-of-concept study	GPT-3.5; 20 (English)	Orthopedic	FKGL and FRE	5

^a^LLM: large language model.

^b^PEM: patient education material.

^c^RGL: reading grade level.

^d^According to the US grade school-level system.

^e^FKGL: Flesch-Kincaid Grade Level.

^f^FRE: Flesch Reading Ease.

^g^LIX: Läsbarhetsindex Index.

^h^RIX: Rate Index.

^i^SMOG: Simple Measure of Gobbledygook.

^j^GPM Fry: Gilliam Peña Mountain Fry Graph.

^k^FHRI: Fernandez-Huerta Readability Index.

^l^SOL: Spanish Orthographic Length.

^m^274 PEMs about neurology, 66 PEMs about neurological surgery.

^n^GFI: Gunning Fog Index.

^o^CLI: Coleman-Liau Index.

^p^LWF: Linsear Write Formula.

^q^ARI: Automated Readability Index.

^r^Excerpts of PEMs.

^s^INFLESZ: Índice Flesch-Szigriszt.

^t^Frequently asked questions.

^u^Extracts were at least 300 words.

^v^Online health information published by recognized national and international health information provider websites.

The geographical distribution showed a strong US dominance (n=27) [36-47,49,51-57,59-65], with additional contributions from the United Kingdom [50], Australia [48], the Netherlands [66], and a US-Thailand collaboration [58]. This geographic focus is reflected in the languages studied, with 30 studies examining English PEMs [36-39,41-66], 2 evaluating both English and Spanish PEMs [37,47], and 1 focusing solely on Spanish PEMs [40].

Sample sizes varied considerably, ranging from 7 to 340 PEMs per study, including full texts, text excerpts [45,48], and frequently asked question sections [58].

**Figure 2:**
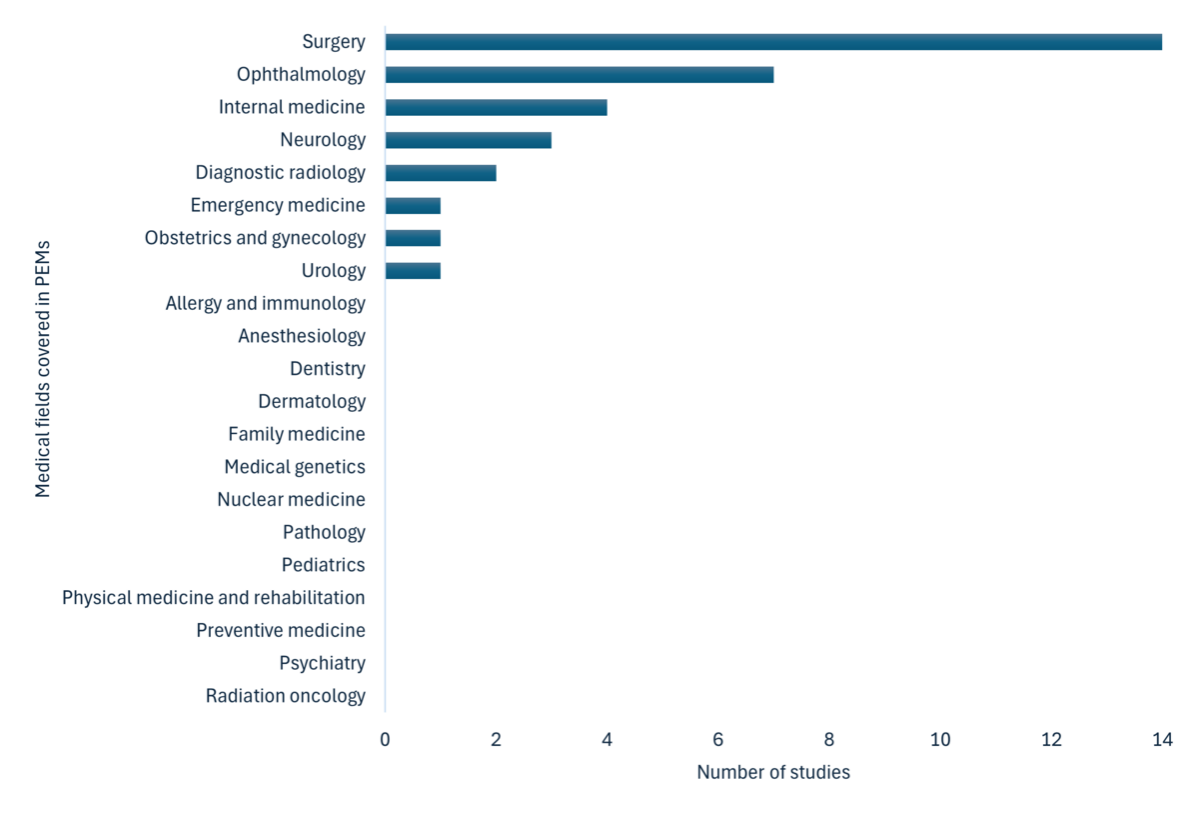
Distribution of medical fields represented in patient education materials across the included studies.

Figure 2 illustrates the distribution of medical fields addressed in the PEMs in the included studies. Surgery was the most frequently investigated field (n=14), encompassing orthopedics, neurosurgery, plastic surgery, oral and maxillofacial surgery, craniofacial surgery, and otolaryngology. Ophthalmology was the second most common field (n=7), covering topics such as glaucoma, cataract, uveitis, and dry eye disease. Additional fields included internal medicine (n=4; heart disease, aortic stenosis, cancer), neurology (n=3; general neurology, stroke, idiopathic intracranial hypertension), and diagnostic radiology (n=2). Single studies addressed emergency medicine (burns first aid) [50], obstetrics and gynecology (reproductive genetics) [66], and urology (renal transplantation) [58]. No studies were identified in the following medical fields: allergy and immunology, anesthesiology, dermatology, dentistry, family medicine, medical genetics, nuclear medicine, pathology, pediatrics, physical medicine and rehabilitation, preventive medicine, psychiatry, or radiation oncology.

To evaluate the effect of prompt engineering, 1 study [59] compared the outputs of GPT-3.5 and GPT-4.0 using 2 different prompts. The first was a simple prompt with a general instruction to simplify the text while maintaining its structure. The second, more detailed prompt provided explicit constraints, referencing specific readability scales and including examples to guide the model’s output [59]. All prompts used in the included studies are presented in Multimedia Appendix 6.

A comprehensive overview of the reported outcomes across all included studies is provided in Multimedia Appendix 7.

### Linguistic Quality

#### Assessment of Linguistic Comprehensibility Versus Absence of Linguistic Correctness Evaluation

All 31 included studies assessed the linguistic quality of simplified PEMs using at least one indicator of linguistic comprehensibility. Notably, none of the studies evaluated linguistic correctness, such as grammatical accuracy or typographical errors.

#### Linguistic Comprehensibility

##### Readability

All included studies assessed linguistic comprehensibility using at least one readability scale. The most frequently used scales for English-language PEMs were the FKGL (n=23 studies) [38,42-44,46,47,49-60,62-66], Flesch Reading Ease (FRE; n=18) [36-38,41-43,46,47,49,50,52,57,59,60,63-66], and Simple Measure of Gobbledygook (SMOG; n=17) [37,39,43,44,48,50-52,54-56,59,62-66]. Additional metrics included the Gunning Fog Index (GFI; n=12) [41,43,44,47,50,52,59,60,62,63,65,66], Coleman-Liau Index (CLI; n=4) [44,50,64,66], Automated Readability Index (ARI; n=3) [41,52,64], Fry score (n=3) [37,59,62], and single-use scales such as the Raygor Estimate, Rate Index (RIX) [59], Läsbarhetsindex (LIX) [37], and Linsear Write Formula (LWF) [66]. Two studies used a composite readability scoring system based on the average of 7 readability indices (FRE, FKGL, SMOG, GFI, CLI, ARI, and LWF) [45,61]. Several studies employed multiple readability scales for comparative analysis. Table 2 provides an overview of the readability scales used to assess English-language PEMs in the included studies, along with their underlying scoring components used to measure readability.

**Figure 3:**
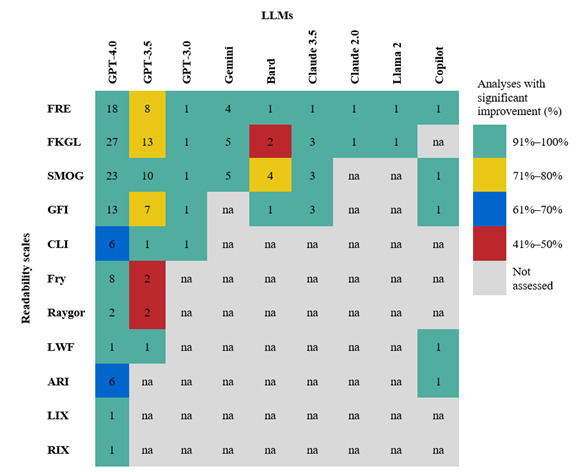
Map analysis of significant readability improvements in English-language patient education materials across all studies, achieved using various large language models and assessed with different readability scales. ARI: Automated Readability Index; CLI: Coleman-Liau Index; FKGL: Flesch-Kincaid Grade Level; FRE: Flesch Reading Ease; GFI: Gunning Fog Index; LIX: Läsbarhetsindex Index; LWF: Linsear Write Formula; RIX: Rate Index; SMOG: Simple Measure of Gobbledygook.

As Figure 3 shows, readability improvement varied by LLM and readability scale in English-language PEMs. GPT-4.0, the most frequently evaluated model, demonstrated the most consistent performance, with a proportion of analyses showing readability improvement falling into the “91%-100%” category across all metrics, except CLI and ARI (67%). By contrast, GPT-3.5 showed more variable results: a 100% success rate on SMOG but lower rates of improvement for FKGL (77%), FRE (75%), and GFI (71%). Other LLMs (GPT-3.0, Gemini, Bard, Claude, Llama 2, and Copilot) also achieved high proportions of analyses showing significant improvements; however, evidence remains limited to single studies for most models.

For Spanish-language PEMs, 3 studies [37,40,47] employed language-specific readability scales such as the Fernández-Huerta Readability Index, GPM Fry Graph, LIX, RIX, Spanish Orthographic Length (SOL), Crawford Nivel-de-Grado, Gutiérrez, Szigriszt-Pazos/Índice Flesch-Szigriszt (INFLESZ), and Legibilidad-μ, achieving improvements in 9 out of 12 (75%) analyses.

**Table 2:** Readability scales used in included studies and corresponding scoring components [67,68].

Scale	Components to score readability
Flesch-Kincaid Grade Level	Average number of syllables per word Average number of words per sentence
Flesch Reading Ease	Average number of syllables Average number of words per sentence Average number of sentences
Simple Measure of Gobbledygook	Average number of words with ≥3 syllables Average number of sentences
Gunning Fog Index	Number of sentences Number of words Number of words with ≥3 syllables
Coleman-Liau Index	Average number of letters per 100 words Average number of sentences per 100 words
Automated Readability Index	Average number of characters per word (eg, any letters, numbers, symbols) Average number of words per sentence
Fry	Average number of sentences Syllables per 100 words
Raygor Estimate	Average number of sentences Long words (≥6 characters) per 100 words
Rate Index	Number of long words Number of sentences
Läsbarhetsindex Index	Average number of words per sentence Percentage of words with >6 letters
Linsear Write Readability	Number of easy words (words with ≤2 syllables) Number of hard words (words with ≥3 syllables)

##### Reading Grade Levels

**Figure 4:**
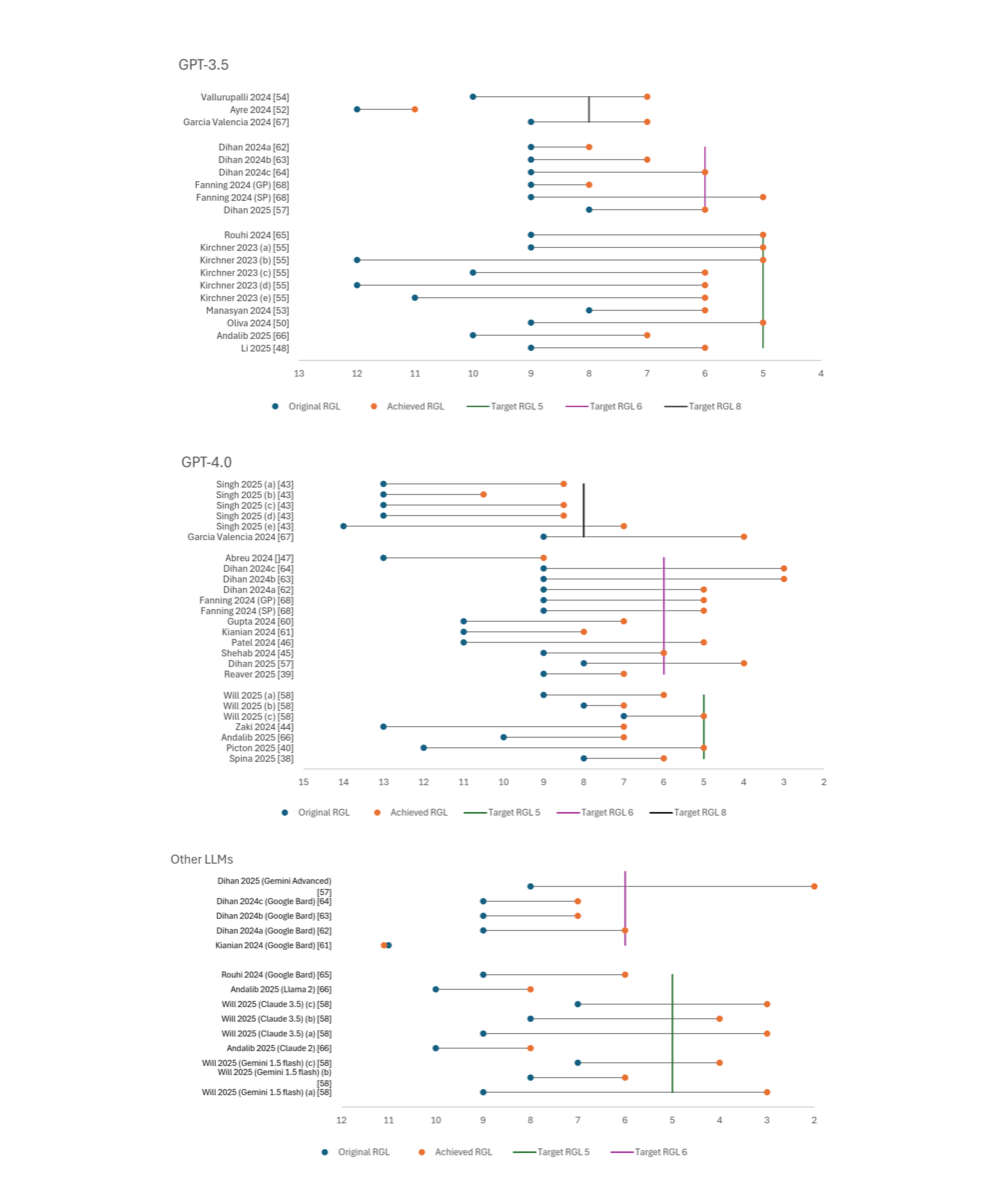
Comparison of original and achieved RGL after prompting various LLMs to simplify PEMs to a predefined target RGL. LLMs: large language models, PEM: patient education material, RGL: reading grade level.

In addition to objective measurements of readability, nearly all included studies (n=30) investigated whether LLMs could achieve predefined target reading grade levels (RGLs) specified in the prompts, typically ranging from fifth to eighth grade. The detailed original, target, and achieved RGLs after prompting LLMs to rewrite PEMs to a specific RGL across all included studies can be found in Figure 4 (also see [38-40,42-46,48-52,54-59,61-63,66-68]).

As Figure 4 shows, the studies demonstrated variable success rates for GPT models. At the fifth-grade target level, GPT-3.5 and GPT-4.0 demonstrated their lowest performance, achieving success in 4 out of 10 (40%) and 2 out of 7 (29%) attempts, respectively. Performance improved notably at the sixth-grade level, where GPT-3.5 reached the target in 3 out of 6 (50%) cases, whereas GPT-4.0 showed success in 8 out of 12 (67%) cases. At the eighth-grade level, an inverse pattern emerged: GPT-3.5 achieved a success rate of over 65% (2/3, 67%), whereas GPT-4.0 succeeded in only <35% of attempts (2/6, 33%).

Among other LLMs, Gemini demonstrated the strongest performance, with an 83% success rate, followed by Claude models at 75%. Conversely, Bard exhibited notably poor performance, achieving target RGLs in only 20% of attempts. Evidence for GPT-3.0 and Llama 2 is limited to single studies, precluding meaningful comparison.

Three studies explored flexible target ranges (sixth to eighth grade) rather than fixed targets, with only 2 of 6 analyses achieving the sixth-grade level recommended by the AMA [39,45,66].

Analysis of Spanish-language PEMs achieving specific RGLs showed mixed results: 1 study successfully reduced readability from sixth to fifth grade using GPT-4.0 [40], whereas another achieved only a minimal reduction (ninth to eighth grade) despite targeting the sixth grade [37]. The third study did not report a specific target RGL [52].

##### Other Linguistic Comprehensibility Indicators

The ability to reduce *text length* was assessed through word count in 14 studies [37,38,40,42,48,51,52,54-57,60,65,66] and sentence count in 5 studies [51,54,55,57,66]. GPT-3.5 reduced word count in all 8 analyses, and GPT-4.0 achieved reductions in nearly 80% of cases (15/19, 79%). Both models maintained readability improvements alongside text reduction in most instances: GPT-3.5 in 75% and GPT-4.0 in 89%. Other LLMs, such as Bard, Claude (2.0 and 3.5), Llama 2, and Copilot, similarly achieved word count reductions while maintaining readability. Sentence count decreased in 13 out of 17 (76%) analyses, with no notable differences between LLMs.

Lexical complexity was assessed in 8 studies [37,51,54-57,60,62]. All tested LLMs (GPT-3.5, GPT-4.0, Bard, Gemini, and Llama 2) reduced syllable-based metrics (polysyllabic words, syllables per word, and syllables per sentence), except Claude 2 in 1 analysis [57]. GPT-4.0 also reduced the number of words exceeding 6 characters [62].

Syntactic complexity was assessed in 3 studies [37,62,66]. Multiple LLMs (GPT-3.5, GPT-4.0, Gemini, and Copilot) successfully reduced long sentences (>20-22 words) and passive voice usage.

Vocabulary complexity was assessed in 2 studies. LLMs effectively simplified vocabulary, reducing medical jargon, acronyms, uncommon words, and complex words [48,66].

Spanish-language studies also confirmed reductions in word count, syllable count, and long sentence frequency [37,40].

### Content Fidelity

#### Identified Studies

A total of 20 studies examined whether the simplified texts remained factually correct and complete [36-38,40,43,46,48,49,51,52,54,55,57,58,61-66].

#### Factual Correctness

Factual correctness was assessed through content similarity in 6 studies [36-38,57,58,62] and content accuracy in 16 studies [37,38,40,43,46,49,51,54,55,58,61-66].

*Content similarity* was evaluated through automated and human assessment methods. Automated analyses using latent semantic analysis with cosine similarity values consistently demonstrated high semantic preservation. GPT-4.0 showed the strongest evidence base, producing “near identical” outputs in all 4 analyses [36,37,57,62], with 3 additional studies reporting “high similarity” [37,38,58]. GPT-3.5, Claude 2, and Llama 2 each achieved “near identical” similarity in single analyses [57]. Expert evaluations, conducted by either research team members or specialized health care professionals, further supported these findings. Human raters consistently confirmed that simplified texts did not contain extraneous information across all tested LLMs [57]. One study reporting quantitative expert assessment of GPT-4.0 yielded a mean similarity score of 0.72 on a 0-1 scale (where 1 represents identical content) [38].

*Content accuracy* was generally high but varied across studies. Automated *F*_1_-scores, which reflect precision and recall (ie, whether a statement is true or false and whether it is present or absent), ranged from 72% to 92% for GPT-4.0 across different studies [37,38,62]. Expert assessments were inconsistent. For GPT-4.0, 8 studies reported no factual errors [43,51,53-55,58,64,65], 1 reported 90%-100% accuracy across 3 raters [52], and 2 studies using 5-point Likert scales reported accuracy between 3.55 and 4.1 (5=completely accurate) [38,66]. One notable outlier reported only 52% factual correctness [40]. For GPT-3.5, several studies reported no factual inaccuracies [46,49,51,54,55,61,63], and where a 5-point Likert scale was used, it scored 3.8 [66]. Other LLMs showed similar variability: Gemini received expert accuracy or suitability ratings of 48%-71% (with up to 14% judged inaccurate) [52], whereas other studies reported error-free outputs [51,65] or 10% inaccuracy rates [65]. Claude showed 5% inaccuracy in some analyses; Copilot received a mean score of 3.51; and Bard achieved 88.9% accuracy and suitability in 1 study [53] while being reported as error-free in other studies [54,55,63].

#### Factual Completeness

Factual completeness was assessed through content retention [43,46,48,49,52,66] and content omission [66] in fewer studies.

*Content retention* varied considerably across the evaluated LLMs. GPT-4.0 demonstrated the most robust performance when compared with GPT-3.5, Gemini, and Copilot [66]. Furthermore, GPT-4.0 maintained sufficient detail with some redundancies (eg, chronic cough was mentioned as a symptom of bronchitis 4 times) [43], and 95% of its outputs retained at least 75% of information [52]. Conversely, Gemini demonstrated reduced retention, with only 68% of outputs achieving the same retention threshold [52]. GPT-3.5 showed inconsistent retention, with studies reporting sufficient detail preservation [49], 80% average content retention [48], or truncation issues in 9% of outputs [46].

*Content omission* was evaluated in 1 study [66]. GPT-4.0 achieved the highest ratings for preserving essential information while simplifying, followed by Copilot. Conversely, Gemini received the lowest scores [66].

### Overview of Text Quality Indicator Assessment Methods

**Figure 5:**
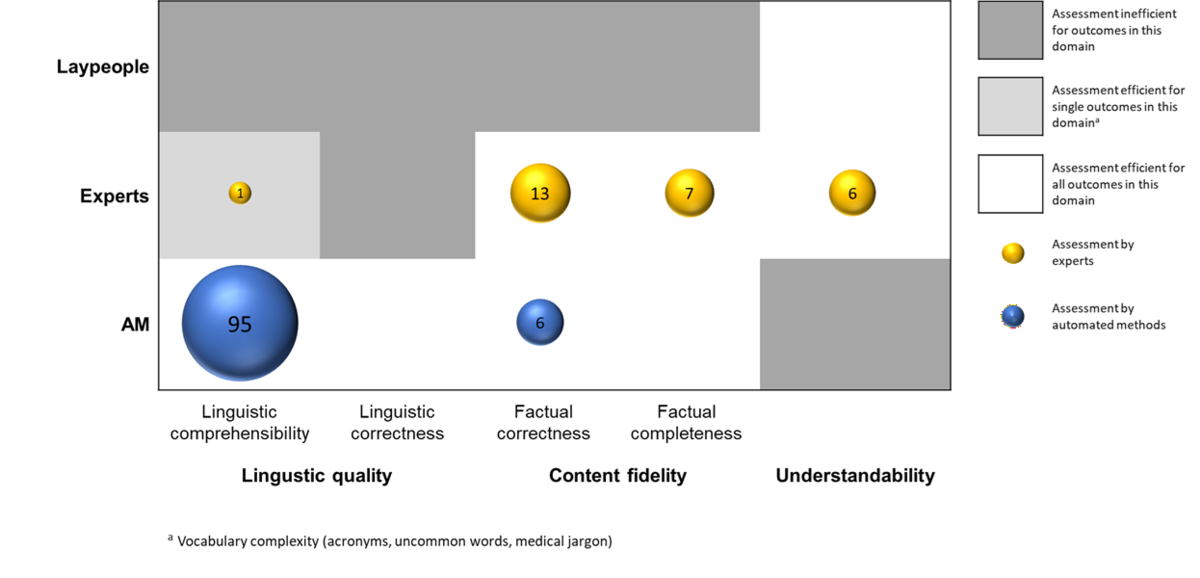
Evidence gap map of assessment methods used across studies for outcome domains (linguistic quality, content fidelity, and understandability) of simplified patient education materials, stratified by assessment type (laypeople, experts, and automated methods). AM: automated method.

As highlighted in Figure 5, linguistic comprehensibility was predominantly assessed using automated methods, as the manual assessment of objective indicators such as readability, sentence length, or word count is inefficient. By contrast, verifying factual correctness and completeness, as well as evaluating the understandability of simplified PEMs, inherently requires human judgment and cannot be fully automated. While content fidelity of simplified PEMs in the included studies was assessed by experts, this occurred less frequently overall. Crucially, the analysis reveals 2 major validation gaps: first, no study assessed the linguistic correctness of the simplified PEMs. Second, and most notably, no study involved laypeople in assessing the actual understandability of the simplified PEMs.

### Relationship Between Readability, Content Accuracy, and Understandability of Simplified PEMs

A total of 6 studies evaluated the understandability and clarity of simplified PEMs through human expert assessments, including research team members or health care professionals, using validated tools such as the Patient Education Materials Assessment Tool [51,54,55,65] and subjective assessments of whether the PEM was understandable for an average patient or successfully improved accessibility without losing key information [52,58]. However, only 4 studies directly examined the relationship between readability improvement, content accuracy, and overall expert-rated understandability of simplified PEMs [54,55,58,65] (Table 3).

**Table 3:** Relationship between readability, content accuracy, and understandability.

Large language model	Readability improvement^a^, n/N (%)	Content accuracy maintenance^b^, n/N (%)	Understandability success^c^, n/N (%)	Medical topic	Studies
GPT-4.0	9/9 (100)	6/6 (100)	3/6 (50)	✓ ^d^Childhood glaucoma, cancer, and living kidney donation ✗ ^e^Idiopathic intracranial hypertension, heart disease, and stroke	[54,55,58,65]
GPT-3.5	3/4 (75)	2/2 (100)	0/2 (0)	✗ Idiopathic intracranial hypertension and childhood glaucoma	[54,55]
Bard	4/4 (100)	2/2 (100)	0/2 (0)	✗ Idiopathic intracranial hypertension and childhood glaucoma	[54,55]
Gemini 1.5	4/4 (100)	2/3 (67)	1/3 (33)	✓ Cancer ✗ Heart disease and stroke	[65]
Claude 3.5	4/4 (100)	1/3 (33)	1/3 (33)	✓ Heart disease ✗ Cancer and stroke	[65]

^a^Number of analyses with significant improvement in at least one readability metric (Flesch-Kincaid Grade Level, Flesch Reading Ease, Simple Measure of Gobbledygook, or Gunning Fog Index).

^b^Number of analyses where no factual errors were detected.

^c^Judgment by human experts

^d^Understandable.

^e^Not understandable.

The studies confirmed that GPT-3.5, GPT-4.0, Bard, Gemini 1.5, and Claude 3.5 consistently improved readability scores across standard metrics, except for GPT-3.5 using FKGL in 1 analysis [54]. Content accuracy was generally high but varied by LLM and medical field. GPT-3.5, GPT-4.0, and Bard achieved 100% content accuracy across multiple conditions. However, Gemini 1.5 demonstrated 10% inaccuracy for heart disease content while maintaining 100% accuracy for cancer and stroke. Claude 3.5 showed the opposite pattern: 100% accuracy for heart disease but 5% inaccuracy for cancer and stroke. Understandability proved most challenging. GPT-4.0 emerged as the most capable, successfully generating readable, accurate, and understandable materials for childhood glaucoma [55] and kidney donation [62]; however, it failed for idiopathic intracranial hypertension [45]. Other models demonstrated greater limitations: GPT-3.5 and Bard generated readable and accurate outputs that experts deemed not understandable across multiple evaluations. Disease-specific performance varied substantially: GPT-4.0 generated understandable PEMs for cancer but not for cardiovascular conditions, whereas Claude 3.5 achieved understandability only for heart disease content [65].

## Discussion

### Principal Findings

This scoping review synthesized evidence on automated language processing technologies currently used to simplify PEMs into layperson-friendly language. A total of 31 studies met the inclusion criteria and examined LLMs for automatic text simplification. Notably, despite a comprehensive literature search, no studies specifically examined AI-supported writing assistants (eg, DeepL Write) or other AI-supported tools designed for automated text simplification. This scoping review has 3 key findings.

First, LLMs consistently improve readability metrics, with GPT-4.0 demonstrating the most reliable performance in comparison with other LLMs. However, achieving predefined RGLs remains challenging across all LLMs, particularly for GPT models at the lowest target level (fifth grade). Notably, GPT-4.0 does not consistently outperform GPT-3.5; rather, performance varies by target RGL. This indicates that newer models are not automatically better at achieving specific RGLs than older ones. Although Gemini and Claude show higher success rates, limited analyses preclude definitive conclusions. At the current stage of technological development, LLMs struggle to consistently achieve the recommended RGL for optimal lay comprehension, particularly the sixth-grade level recommended by the AMA. However, based on the available data, it remains unclear whether the failure to consistently achieve target RGLs stems from inherent model limitations or from heterogeneity in prompting strategies across the included studies.

Second, content fidelity assessments showed considerable variability. Although content similarity scores were generally high, content accuracy ranged from 48% to 100% across studies. This level of inaccuracy is concerning in medical contexts, where even minor errors can compromise patient safety.

Third, and most critically, this scoping review reveals a fundamental validation gap: no study has evaluated linguistic correctness (eg, grammar or typographical errors), and no study has assessed whether laypeople actually understand the simplified PEMs. The absence of reporting on linguistic correctness indicators suggests that authors implicitly assume that LLM-simplified texts are grammatically correct. However, this assumption can be problematic in medical contexts, where ambiguous phrasing or grammatical errors can alter the meaning of health instructions and potentially cause harm to patients. For instance, even minor errors in negation or word order can result in dangerous misinterpretations. Therefore, the failure to systematically assess and report linguistic correctness represents both a methodological oversight and a patient safety concern. Furthermore, the few studies that assessed overall understandability relied exclusively on expert assessments. However, experts such as health care professionals cannot reliably predict what laypeople understand or do not understand. This validation gap reveals a critical conceptual conflation: readability, as measured by formulas, captures surface-level text characteristics such as sentence length, word length, and syllable counts. However, it does not measure whether a reader actually understands the content. By contrast, understandability refers to the extent to which readers can extract meaning from text, apply the information to their own situation, and make informed decisions based on what they read. Consequently, improved readability metric scores do not guarantee that medical content is also factually correct, contextually appropriate, or genuinely understandable to the target group. This distinction is particularly significant in medical contexts, where patients must not only read but also correctly interpret and act upon health information. The complete absence of patient-centered outcome assessment represents the most significant finding of this scoping review.

### Comparison With Previous Work

To the best of our knowledge, this is the first scoping review focusing specifically on automatic text simplification methods for PEMs. This distinguishes our work from previous research on medical AI, which has predominantly focused on text generation [69-78] rather than text simplification or the simplification of clinical records [79-82], rather than PEMs.

Nguyen et al [83] conducted a systematic review and meta-analysis of online PEMs related to cleft lip and palate. In line with our findings, they identified the critical absence of understandability testing, as only 1 study directly assessed patient understanding of simplified texts. In contrast to their disease-specific approach, our review spans a broader range of medical topics, evaluates linguistic quality and content fidelity, and employs a broader search strategy.

In a broader scoping review, Aydin et al [84] examined LLM applications across multiple domains of patient care, including education, engagement, workload reduction, patient-centered health customization, and communication. However, their search was limited to PubMed and was conducted in June 2024. They similarly reported readability improvements in studies addressing automatic text simplification. Their conclusion that LLMs can create accessible materials, help interpret complex information, and enhance patient-provider communication—while also noting that accuracy, readability issues, and ethical concerns require further development—aligns with the findings of our review.

The complete absence of direct testing of understandability with laypeople in all 31 studies is significant, reflecting a systemic methodological limitation in this field: the uncritical acceptance of readability formulas as valid measures of text simplification. These findings are further supported by studies indicating that standard readability formulas have important limitations. They primarily count variables such as sentence length, word length, or polysyllabic words, but do not assess whether the text aligns with the actual comprehension, context, expertise, semantic understanding, and textual coherence of the target audience. Common readability formulas cannot adequately assess the actual understandability of medical texts [85]. Furthermore, readability formulas may judge short technical terms and abbreviations as simple, despite being far less understandable than longer, everyday descriptions [86,87]. For example, the term “HbA_1c_” is very short but often unknown, whereas the term “long-term blood sugar” is longer but more self-explanatory. Additionally, in-text explanations of terms are often needed for comprehension; however, they can lengthen and complicate sentences [88].

### Research Implications

#### Overview

The findings of this review highlight several research gaps, constituting an urgent call to action for future studies. The validation gap identified in this review—namely, the complete absence of patient-centered comprehension testing and linguistic correctness assessment—should be addressed as a priority in future research. The following research gaps should guide the field forward:

#### Patient-Centered Evaluation

This represents the most significant research gap. Future studies must shift from purely algorithmic readability evaluation to patient-centered understandability testing [37,46,47,55-57,60,62,66]. Future studies should involve laypeople with varying health literacy levels, age, education, and cultural backgrounds in understandability assessments using validated methods, rather than relying solely on expert judgment or readability formulas [44,48,58]. For instance, Ondov et al [89] suggested 2 methods of comprehension assessment:

- Multiple-choice questions: After reading the original or simplified text, users answer constructed items that require understanding of the text to respond correctly. These questions are reliable when developed and validated properly, but are labor-intensive and require domain and assessment expertise.
- Cloze tests: These tests involve deleting words in a passage and asking users to supply the missing terms from context. They correlate well with other comprehension measures and are largely automatable. Common variants include basic cloze (masking important content words) and multiple-choice cloze (using distractors for each blank).

#### Prompt Engineering

Prompt engineering requires systematic investigation. In this scoping review, only 1 study [59] systematically investigated prompt design. Fanning et al [59] compared general and specific prompts using GPT-3.5 and GPT-4.0 and demonstrated that prompt design can markedly influence output quality. The study shows that well-designed, specific prompts enabled GPT-3.5 to achieve readability improvements comparable to GPT-4.0, suggesting that prompt optimization may be more influential than model generation for text simplification tasks. Their specific prompts included explicit references to readability measures, detailed instructions for writing style (eg, shortening sentences and simplifying words), specification of target readability levels, and explicit constraints requiring that rewritten texts maintain the original meaning and information. While these findings are based on a single study, they offer a possible explanation for the inconsistent results observed across the included studies. The wide variability in content accuracy (48%-100%) and frequent failure to achieve specific RGLs could be attributed to differences in prompt design rather than solely to inherent model limitations. However, further research is needed to confirm this. Future studies should establish best practices for prompt engineering [36,37,39,41,42,45,47,51,53,59,61,65]. This involves systematically comparing different prompting strategies. For instance, moving beyond basic zero-shot prompts to incorporate advanced techniques such as few-shot prompting (providing models with examples of desired simplified outputs) [37,65] or chain-of-thought prompting (guiding the model through a step-by-step reasoning process). Chain-of-thought prompting could instruct the LLM to first identify complex medical terms, then suggest layperson equivalents, then shorten sentences, and finally check for factual accuracy [90]. Similarly, exploring the impact of stylistic constraints, detailed context-specific instructions, and the use of word-substitution lists is essential [37]. Further, future studies should focus on iterative prompt design with feedback loops for continuous refinement based on expert and patient evaluations, as well as evaluating prompt stability across LLM generations [47,61]. The reliability of prompts should also be assessed through test-retest consistency, ensuring that identical prompts yield consistent, high-quality outputs over time [91]. Finally, a key long-term goal should be the development of a validated, open-access prompt library with demonstrated efficacy for medical text simplification, enabling standardization across research and clinical applications. While existing technical prompt collections in the medical domain primarily address reasoning and question-answering tasks (eg, Microsoft MedPrompt), they were neither designed nor clinically validated for medical text simplification [92].

#### Technological Advancements

The rapid progress in LLM technology requires continuous evaluation. Future studies should systematically compare emerging LLMs, particularly beyond the GPT series, to identify models with unique strengths (eg, long-context handling with Claude 2) [44,47,48,59,65]. Furthermore, cost-effectiveness comparisons between commercial and open-source LLMs are also needed [47,55,63].

#### Geographic and Linguistic Diversity

The included studies predominantly focused on English-language PEMs within the US health care context, with only 3 studies [37,40,47] evaluating Spanish texts. Consequently, the generalizability of these findings to other languages and cultural health care systems is limited. Languages with complex morphology (eg, German or Slavic languages) or distinct syntactic structures may present different challenges for automated simplification than English. Furthermore, cultural differences in medical communication styles may affect how “layperson-friendly” is defined and achieved in other regions. Therefore, future studies should investigate simplification performance across diverse linguistic and cultural contexts [48,49,62,65,66].

#### Medical Field Scope

The included studies predominantly focused on surgical specialties and ophthalmology, while numerous medical fields remain unexplored with regard to LLM-based text simplification of PEMs. No studies addressed, for example, dentistry, dermatology, psychiatry, or preventive medicine. This is notable, as these fields frequently rely on PEMs containing complex terminology. Dentistry, for example, involves jargon-heavy procedures in areas such as orthodontics that may be difficult for patients to understand [93]. Future research should investigate LLM-based text simplification across a broader range of medical fields, especially rare diseases and less prevalent health conditions, where LLMs with limited training data may be more susceptible to errors [48,65].

#### Linguistic Correctness

None of the included studies assessed text quality indicators related to linguistic correctness. Thus, future evaluations should incorporate metrics for grammar and typographical errors, as these factors are fundamental to establishing credibility and trust, independent of their direct effect on comprehension [94,95].

### Practice Implications

Based on the findings of this scoping review, this study has several recommendations for clinical practice. First, LLMs should be used as assistive rather than autonomous tools for simplifying PEMs used in clinical practice [51]. This implication is supported by a recent study showing that LLMs can approximate targeted RGLs when simplifying health information materials about psychiatry, but their outputs are inconsistent, with significant variability in reading levels and deviations from the intended content, making them unsuitable for standalone deployment in health care settings [96]. Second, all AI-simplified PEMs must undergo mandatory expert review by qualified health care professionals to verify content fidelity and clinical appropriateness before dissemination to laypeople [38-41,45-49,52,59-61,66]. Third, health care organizations using AI-assisted text simplification tools should establish clear protocols defining quality checkpoints and approval workflows. Continuous monitoring of performance can track both readability improvements and error rates across different medical domains [59].

Although LLMs hold considerable promise for enhancing patient health literacy through simplified text versions, their use in clinical practice requires careful attention to inherent limitations, including the risk of reinforcing biases and stereotypes embedded in training data. The use of AI in health care communication requires an ongoing commitment to the accuracy of generated content [50].

### Limitations

Although this scoping review followed rigorous and systematic methods, some limitations must be acknowledged. First, rule-based technologies were excluded from this review. This decision was made to focus on the contemporary and rapidly evolving landscape dominated by generative AI, which offers greater scalability and adaptability for text simplification. Second, although different prompting strategies were used across the included studies, the quality of these prompts was not evaluated. In accordance with scoping review methodology, which aims to map the extent and nature of available evidence rather than critically appraise methodological quality, a systematic assessment of prompt design was not conducted. Consequently, it was not possible to determine whether the observed failure to meet specific target RGLs reflects model limitations or suboptimal prompting strategies. However, such an analysis could have provided deeper insights into optimal prompt engineering for PEM simplification. Lastly, the database search was conducted in May 2025, and the gray literature search in July 2025. Given the rapid pace of AI development, particularly regarding LLMs, new relevant studies may have emerged since, and some findings may already become outdated shortly after publication. Finally, the included studies were heavily skewed toward English-language PEMs and US-based contexts, limiting the generalizability of the findings, as text simplification approaches may perform differently across languages with varying grammatical complexity (eg, morphology, syntax). Moreover, LLMs are predominantly trained on English data [97], which may lead to lower performance in other languages. Additionally, the readability metrics used (eg, US RGLs) are specific to the US educational system and may not directly translate to plain-language guidelines in other cultural health care systems.

### Conclusions

To our knowledge, this is the first scoping review to comprehensively synthesize evidence on automated language processing technologies for PEM simplification, systematically mapping linguistic, quality, and content fidelity outcomes.

The findings of our scoping review indicate that although LLMs can improve the readability of PEMs, they predominantly fail to achieve the recommended sixth-grade RGL. However, the most significant finding is the identification of a critical validation gap: no study has assessed whether laypeople actually understood the simplified PEMs, nor has any study evaluated linguistic correctness. Coupled with variable content accuracy and reliance solely on readability formulas that poorly predict actual understanding by laypeople, these findings have important implications for clinical practice. Currently, LLMs should serve as assistive tools rather than autonomous solutions. All AI-simplified materials must undergo mandatory expert review to verify content fidelity before dissemination to laypeople. Future research must urgently shift from purely algorithmic evaluation to patient-centered validation to directly assess laypeople’s comprehension.

## Appendix

Multimedia Appendix 1 PRISMA-ScR (Preferred Reporting Items for Systematic Reviews and Meta-Analyses extension for Scoping Reviews) checklist.

Multimedia Appendix 2 PRISMA-S (PRISMA Statement for Reporting Literature Searches in Systematic Reviews) checklist.

Multimedia Appendix 3 Search strategies for bibliographic databases.

Multimedia Appendix 4 Excluded studies.

Multimedia Appendix 5 Trend analysis of publication dates for studies evaluating large language models for the text simplification of patient education materials (2019-2025).

Multimedia Appendix 6 Overview of prompts used for automated text simplification in the included studies.

Multimedia Appendix 7 Overview of reported outcomes in included studies.

## Abbreviations

AI: artificial intelligence
AMA: American Medical Association
ARI: Automated Readability Index
BLEU: Bilingual Evaluation Understudy
CLI: Coleman-Liau Index
FKGL: Flesch-Kincaid Grade Level
FRE: Flesch Reading Ease
GFI: Gunning Fog Index
INFLESZ: Índice Flesch-Szigriszt
JBI: Joanna Briggs Institute
LIX: Läsbarhetsindex Index
LLM: large language model
LWF: Linsear Write Formula
NLP: natural language processing
PCC: Population-Concept-Context
PEM: patient education material
PRISMA: Preferred Reporting Items for Systematic Reviews and Meta-Analyses
PRISMA-S: PRISMA Statement for Reporting Literature Searches in Systematic Reviews
PRISMA-ScR: Preferred Reporting Items for Systematic Reviews and Meta-Analyses extension for Scoping Reviews
RGL: reading grade level
RIX: Rate Index
ROUGE: Recall-Oriented Understudy for Gisting Evaluation
SMOG: Simple Measure of Gobbledygook
SOL: Spanish Orthographic Length

## Data Availability

All extracted data relevant to the research aims are included in the manuscript and its multimedia appendices.
