# Supplementary material for: Automated Approaches of Text Simplification of Patient Education Materials: Scoping Review
Source: J Med Internet Res. 2026 May 7;28:e88365. doi: 10.2196/88365 (PMC13195379; doi:10.2196/88365)
Supplement: Multimedia Appendix 3 [file jmir_v28i1e88365_app3.docx]

**Multimedia Appendix 2 - Search strategies**

**Ovid MEDLINE**

|  | **Searches** |
| --- | --- |
| 1 | exp Artificial Intelligence/ |
| 2 | ("artificial intelligence" or "AI" or "machine learning" or "machine intelligence" or "computational intelligence" or "deep learning" or "large language model*" or LLM or "natural language processing*" or NLP or "machine translation*" or "neural machine translation*" or NMT or "translation tool*" or "translation engine*" or "translation system*" or "neural network*" or "generative pretrained transformer" or "generative pre-trained transformer" or "artificial intelligence chatbot" or "GPT" or ChatGPT or "Generative Transformer" or "Generative Language Model" or "Generative Model").ti,ab. |
| 3 | 1 or 2 |
| 4 | ("lay language" or "lay term*" or laywom?n or laypeople or "lay people" or layperson* or "lay person*" or laity or "easy-to-read" or "easy-to-understand" or "easy write" or "easy info").ti,ab. |
| 5 | ((text* or language) adj3 (plain or everyday or simpl* or comprehension or comprehensibl* or comprehend* or understand* or readab* or rewrit* or lay? or patient-friendly or non-technical or easy or aphasia-friendly or clear or accessible or user-friendly or common or basic)).ti,ab. |
| 6 | (text* or language).ti,ab. and comprehension/ |
| 7 | language/ and (plain or everyday or simpl* or comprehension or comprehensibl* or comprehend* or understand* or readab* or rewrit* or lay? or patient-friendly or non-technical or easy or aphasia-friendly or clear or accessible or user-friendly or common or basic).ti,ab. |
| 8 | language/ and comprehension/ |
| 9 | 4 or 5 or 6 or 7 or 8 |
| 10 | 3 and 9 |
| 11 | limit 10 to (english language and yr="2019 -Current" and "remove preprint records") |

**Embase**

|  | **Searches** |
| --- | --- |
| 1 | exp artificial intelligence/ |
| 2 | machine learning/ |
| 3 | deep learning/ |
| 4 | large language model/ |
| 5 | natural language processing/ |
| 6 | exp generative pretrained transformer/ |
| 7 | exp artificial intelligence chatbot/ |
| 8 | ("artificial intelligence" or "AI" or "machine learning" or "machine intelligence" or "computational intelligence" or "deep learning" or "large language model*" or LLM or "natural language processing*" or NLP or "machine translation*" or "neural machine translation*" or NMT or "translation tool*" or "translation engine*" or "translation system*" or "neural network*" or "generative pretrained transformer" or "generative pre-trained transformer" or "artificial intelligence chatbot" or "GPT" or ChatGPT or "Generative Transformer" or "Generative Language Model" or "Generative Model").ti,ab. |
| 9 | 1 or 2 or 3 or 4 or 5 or 6 or 7 or 8 |
| 10 | plain language/ |
| 11 | ("lay language" or "lay term*" or laywom?n or laypeople or "lay people" or layperson* or "lay person*" or laity or "easy-to-read" or "easy-to-understand" or "easy write" or "easy info").ti,ab. |
| 12 | ((text* or language) adj3 (plain or everyday or simpl* or comprehension or comprehensibl* or comprehend* or understand* or readab* or rewrit* or lay? or patient-friendly or non-technical or easy or aphasia-friendly or clear or accessible or user-friendly or common or basic)).ti,ab. |
| 13 | (text* or language).ti,ab. and comprehension/ |
| 14 | language/ and (plain or everyday or simpl* or comprehension or comprehensibl* or comprehend* or understand* or readab* or rewrit* or lay? or patient-friendly or non-technical or easy or aphasia-friendly or clear or accessible or user-friendly or common or basic).ti,ab. |
| 15 | comprehension/ and language/ |
| 16 | 10 or 11 or 12 or 13 or 14 or 15 |
| 17 | 9 and 16 |
| 18 | limit 17 to (english language and "remove preprint records" and yr="2019 -Current") |

**PsycInfo**

|  | **Searches** |
| --- | --- |
| 1 | exp artificial intelligence/ |
| 2 | machine translation/ |
| 3 | ("artificial intelligence" or "AI" or "machine learning" or "machine intelligence" or "computational intelligence" or "deep learning" or "large language model*" or LLM or "natural language processing*" or NLP or "machine translation*" or "neural machine translation*" or NMT or "translation tool*" or "translation engine*" or "translation system*" or "neural network*" or "generative pretrained transformer" or "generative pre-trained transformer" or "artificial intelligence chatbot" or GPT or ChatGPT or "generative transformer" or "generative language model" or "generative model").ti,ab. |
| 4 | 1 or 2 or 3 |
| 5 | ("lay language" or "lay term*" or laywom?n or laypeople or "lay people" or layperson* or "lay person*" or laity or "easy-to-read" or "easy-to-understand" or "easy write" or "easy info").ti,ab. |
| 6 | ((text* or language) adj3 (plain or everyday or simpl* or comprehension or comprehensibl* or comprehend* or understand* or readab* or rewrit* or lay? or rewrit* or patient-friendly or non-technical or easy or aphasia-friendly or clear or accessible or user-friendly or common or basic)).ti,ab. |
| 7 | (text* or language).ti,ab. and (comprehension/ or readability/) |
| 8 | language/ and (plain or everyday or simpl* or comprehension or comprehensibl* or comprehend* or understand* or readab* or rewrit* or lay? or rewrit* or patient-friendly or non-technical or easy or aphasia-friendly or clear or accessible or user-friendly or common or basic).ti,ab. |
| 9 | language/ and (comprehension/ or readability/) |
| 10 | 5 or 6 or 7 or 8 or 9 |
| 11 | 4 and 10 |
| 12 | limit 11 to (english language and yr="2019 -Current") |

**CINAHL**

|  | **Searches** |
| --- | --- |
| S1 | MH "Artificial Intelligence+" |
| S2 | XB (“artificial intelligence" or “AI” or "machine learning" or "machine intelligence" or “machine intelligence” or “computational intelligence” or "deep learning" or "large language model*" or LLM or "natural language processing*" or NLP or "machine translation*" or "neural machine translation*" or NMT or "translation tool*" or "translation engine*" or "translation system*" or “neural network*” or "generative pretrained transformer" or "generative pre-trained transformer" or "artificial intelligence chatbot" or "GPT" or ChatGPT or "Generative Transformer" or "Generative Language Model" or "Generative Model") |
| S3 | 1 or 2 |
| S4 | XB ("lay language" or "lay term*" or laywom?n or laypeople or "lay people" or layperson* or "lay person*" or laity or "easy-to-read" or "easy-to-understand" or "easy write" or "easy info") |
| S5 | (XB (text* or language)) N3 (XB (plain or everyday or simpl* or comprehension or comprehensibl* or comprehend* or understand* or readab* or rewrit* or lay? or patient-friendly or non-technical or easy or aphasia-friendly or clear or accessible or user-friendly or common or basic)) |
| S6 | (MH "language+") AND XB (plain or everyday or simpl* or comprehension or comprehensibl* or comprehend* or understand* or readab* or rewrit* or lay? or patient-friendly or non-technical or easy or aphasia-friendly or clear or accessible or user-friendly or common or basic) |
| S7 | MH "language+" AND MH "Readability" |
| S8 | XB (text* or language) AND MH "Readability" |
| S9 | 4 or 5 or 6 or 7 or 8 |
| S10 | 3 and 9 |
| S11 | limit 10 to (english language and “remove medline records” and yr=”2019 -Current”) |

**IEEE Xplore**

|  | **Searches** |
| --- | --- |
| 1 | ("Index Terms":Generative AI) OR ("Index Terms":Report Simplifier) OR ("Index Terms":Automatic System) OR ("Index Terms":Artificial Intelligence Models) OR ("Index Terms":Machine Learning) OR ("Index Terms":Large Language Models) OR ("Index Terms":Natural Language Processing Models) OR ("Index Terms":Natural Language Processing Techniques) OR ("Index Terms":Machine Learning Models) OR ("Index Terms":Natural Language) OR ("Index Terms":machine intelligence) OR ("Index Terms":Deep learning) OR ("Index Terms":machine translation) OR ("Index Terms":neural network) OR ("Index Terms":generative pretrained transformer) OR ("Index Terms":generative pre-trained transformer) OR ("Index Terms":artificial intelligence chatbot) OR ("Index Terms":ChatGPT) OR ("Index Terms":GPT) |
| 2 | ("Index Terms":Medical Information) OR ("Index Terms":Healthcare Information) OR ("Index Terms":Medical Reports) OR ("Index Terms":Medical Text) OR ("Index Terms":Medical Jargon) OR ("Index Terms":Medical Terms) OR ("Index Terms":Medical Interpretation) OR ("Index Terms":Medical Literature) OR ("Index Terms":Electronic Health Records) OR ("Index Terms":[Radiology Reports](https://ieeexplore.ieee.org/search/searchresult.jsp?matchBoolean=true&queryText=%22Index%20Terms%22:Radiology%20Reports&newsearch=true)) OR ("Index Terms":Health-related Text) OR ("Index Terms":Health Information) OR ("Index Terms":Understand Health Information) OR ("Index Terms":Biomedical Abstracts) OR ("Index Terms":PubMed Abstracts) OR ("Index Terms":Medical Context) OR ("Index Terms":Online Health Information) OR ("Index Terms":Patient Education) OR ("Index Terms":Understand Health Information) OR ("Index Terms":Health Education) |
| 3 | ("Index Terms":Reading Level) OR ("Index Terms":Flesch-Kincaid Grade Level) OR ("Index Terms":Simplified Version) OR ("Index Terms":Grade Level) OR ("Index Terms":Simple Text) OR ("Index Terms":Flesch Reading Ease) OR ("Index Terms":Plain English) OR ("Index Terms":Language Barriers) OR ("Index Terms": Plain Language) OR ("Index Terms":Text Comprehension) OR ("Index Terms":Reading Comprehension) OR ("Index Terms":Comprehensive Information) OR ("Index Terms":Simple Terms) OR ("Index Terms":Readability Of The Text) |
| 4 | 1 AND 2 AND 3 |
| 5 | limit to 2019-2025 |
