# Supplementary material for: Automated Approaches of Text Simplification of Patient Education Materials: Scoping Review
Source: J Med Internet Res. 2026 May 7;28:e88365. doi: 10.2196/88365 (PMC13195379; doi:10.2196/88365)
Supplement: Multimedia Appendix 4 [file jmir_v28i1e88365_app4.docx]

**Multimedia Appendix 3 - List of excluded studies**

| **Reference** | **Reason for exclusion** |
| --- | --- |
| Cohen T, Xu W, Guo Y, Pakhomov S, Leroy G. Coherence and comprehensibility: Large language models predict lay understanding of health-related content. Journal of Biomedical Informatics. 2025;161:104758. PMID: 2036334454. doi: https://dx.doi.org/10.1016/j.jbi.2024.104758. | No automatic text simplification |
| Yuce A, Yerli M, Misir A, Cakar M. Enhancing patient information texts in orthopaedics: How OpenAI's 'ChatGPT' can help. Journal of Experimental Orthopaedics. 2024;11(3):e70019. PMID: 2031383334. doi: https://dx.doi.org/10.1002/jeo2.70019. | No automatic text simplification |
| Tang CC, Nagesh S, Fussell DA, Glavis-Bloom J, Mishra N, Li C, et al. Generating colloquial radiology reports with large language models. Journal of the American Medical Informatics Association. 2024;31(11):2660 EP - 7. PMID: 2035189304. doi: https://dx.doi.org/10.1093/jamia/ocae223. | No automatic text simplification |
| Swanson K, He S, Calvano J, Chen D, Telvizian T, Jiang L, et al. Biomedical text readability after hypernym substitution with fine-tuned large language models. PLOS digital health. 2024;3(4):e0000489. doi: https://dx.doi.org/10.1371/journal.pdig.0000489. | No automatic text simplification |
| Rahman MM, Irbaz MS, North K, Williams MS, Zampieri M, Lybarger K. Health text simplification: An annotated corpus for digestive cancer education and novel strategies for reinforcement learning. Journal of Biomedical Informatics. 2024;158:104727. PMID: 2034621037. doi: https://dx.doi.org/10.1016/j.jbi.2024.104727. | No automatic text simplification |
| Kim DY, Lym HJ, Lee H, Lee YJ, Kim J, Kim MG, et al. Child-Centric Robot Dialogue Systems: Fine-tuning large language models for better utterance understanding and interaction. Sensors (Basel, Switzerland). 2024;24(24). PMID: 646248807. doi: https://dx.doi.org/10.3390/s24247939. | No automatic text simplification |
| Khanna P, Dhillon G, Buddhavarapu V, Verma R, Kashyap R, Grewal H. Artificial intelligence in multilingual interpretation and radiology assessment for clinical language evaluation (AI-MIRACLE). Journal of Personalized Medicine. 2024;14(9):923. PMID: 2031621207. doi: https://dx.doi.org/10.3390/jpm14090923. | No automatic text simplification |
| Hershenhouse JS, Mokhtar D, Eppler MB, Rodler S, Storino Ramacciotti L, Ganjavi C, et al. Accuracy, readability, and understandability of large language models for prostate cancer information to the public. Prostate Cancer and Prostatic Diseases. 2024. PMID: 2029837930. doi: https://dx.doi.org/10.1038/s41391-024-00826-y. | No automatic text simplification |
| Grunebaum A, Dudenhausen J, Chervenak FA. Enhancing patient understanding in obstetrics: The role of generative AI in simplifying informed consent for labor induction with oxytocin. Journal of Perinatal Medicine. 2024. PMID: 2035507329. doi: https://dx.doi.org/10.1515/jpm-2024-0428. | No automatic text simplification |
| Minutolo A, Damiano E, De Pietro G, Fujita H, Esposito M. A conversational agent for querying Italian patient information leaflets and improving health literacy. Computers in Biology and Medicine. 2022;141:105004. PMID: 2015525900. doi: https://dx.doi.org/10.1016/j.compbiomed.2021.105004. | No automatic text simplification |
| Oztermeli AD. Is ChatGPT a reliable tool for explaining medical terms? Cureus. 2025;17(1):e77258. doi: https://dx.doi.org/10.7759/cureus.77258. | Automatic text generation |
| Mondal H, Gupta G, Sarangi PK, Sharma S, Choudhary PK, Juhi A, et al. Assessing the capability of large language model chatbots in generating plain language summaries. Cureus. 2025;17(3):e80976. doi: https://dx.doi.org/10.7759/cureus.80976. | Automatic text generation |
| Elhakim T, Brea AR, Fidelis W, Paravastu SS, Malavia M, Omer M, et al. Enhanced PROcedural information READability for patient-centered care in interventional radiology with large language models (PRO-READ IR). Journal of the American College of Radiology : JACR. 2025;22(1):84-97. doi: https://dx.doi.org/10.1016/j.jacr.2024.08.010. | Automatic text generation |
| Stanceski K, Zhong S, Zhang X, Khadra S, Tracy M, Koria L, et al. The quality and safety of using generative AI to produce patient-centred discharge instructions. npj Digital Medicine. 2024;7(1):329. PMID: 2032330502. doi: https://dx.doi.org/10.1038/s41746-024-01336-w. | Automatic text generation |
| Srinivasan N, Samaan JS, Rajeev ND, Kanu MU, Yeo YH, Samakar K. Large language models and bariatric surgery patient education: a comparative readability analysis of GPT-3.5, GPT-4, Bard, and online institutional resources. Surgical endoscopy. 2024;38(5):2522-32. doi: https://dx.doi.org/10.1007/s00464-024-10720-2. | Automatic text generation |
| Postaci SA, Dal A. The ability of large language models to generate patient information materials for retinopathy of prematurity: Evaluation of readability, accuracy, and comprehensiveness. Turkish Journal of Ophthalmology. 2024;54(6):330 EP - 6. PMID: 2035621797. doi: https://dx.doi.org/10.4274/tjo.galenos.2024.58295. | Automatic text generation |
| Musheyev D, Pan A, Gross P, Kamyab D, Kaplinsky P, Spivak M, et al. Readability and information quality in cancer information from a free vs paid chatbot. JAMA Network Open. 2024;7(7):e2422275-e. doi: 10.1001/jamanetworkopen.2024.22275. | Automatic text generation |
| Kilinc DD, Mansiz D. Examination of the reliability and readability of chatbot generative pretrained transformer's (ChatGPT) responses to questions about orthodontics and the evolution of these responses in an updated version. American journal of orthodontics and dentofacial orthopedics: official publication of the American Association of Orthodontists, its constituent societies, and the American Board of Orthodontics. 2024;165(5):546 EP - 55. PMID: 644105494. doi: https://dx.doi.org/10.1016/j.ajodo.2023.11.012. | Automatic text generation |
| Valentin-Bravo FJ, Mateos-Alvarez E, Usategui-Martin R, Andres-Iglesias C, Pastor-Jimeno JC, Pastor-Idoate S. Artificial intelligence and new language models in ophthalmology: Complications of the use of silicone oil in vitreoretinal surgery. Archivos de la Sociedad Espanola de Oftalmologia. 2023;98(5):298-303. doi: https://dx.doi.org/10.1016/j.oftale.2023.04.011. | Automatic text generation |
| Oztermeli AD. Is ChatGPT a reliable tool for explaining medical terms? Cureus. 2025;17(1):e77258. doi: https://dx.doi.org/10.7759/cureus.77258. | Automatic text generation |
| Abdelgadir YH, Thongprayoon C, Craici IM, Cheungpasitporn W, Miao J. Improving patient understanding of glomerular disease terms with ChatGPT. International Journal of Clinical Practice. 2025;2025(1):9977290. PMID: 2033551703. doi: https://dx.doi.org/10.1155/ijcp/9977290. | Text simplification of no health information |
| Li Z, Belkadi S, Micheletti N, Han L, Shardlow M, Nenadic G, editors. Investigating large language models and control mechanisms to improve text readability of biomedical abstracts. 2024 IEEE 12th International Conference on Healthcare Informatics (ICHI); 2024 3-6 June 2024. IEEE Xplore. | Text simplification of no health information |
| Garcia-Rudolph A, Sanchez-Pinsach D, Wright MA, Opisso E, Vidal J. Assessing readability of explanations and reliability of answers by GPT-3.5 and GPT-4 in non-traumatic spinal cord injury education. Medical teacher. 2025:1 EP - 8. PMID: 646363451. doi: https://dx.doi.org/10.1080/0142159X.2024.2430365. | Health information not targeted to laypeople |
| Balas M, Kaplan AJ, Esmail K, Saleh S, Sharma RA, Yan P, et al. Translating ophthalmic medical jargon with artificial intelligence: a comparative comprehension study. Canadian Journal of Ophthalmology. 2025. PMID: 2036791927. doi: https://dx.doi.org/10.1016/j.jcjo.2024.11.003. | Health information not targeted to laypeople |
| Dergaa I, Ben Saad H, Glenn JM, Ben Aissa M, Taheri M, Swed S, et al. A thorough examination of ChatGPT-3.5 potential applications in medical writing: A preliminary study. Medicine (United States). 2024;103(40):e39757. PMID: 2034968996. doi: https://dx.doi.org/10.1097/MD.0000000000039757. | Health information not targeted to laypeople |
| Abdulnazar A, Roller R, Schulz S, Kreuzthaler M. Large language models for clinical text cleansing enhance medical concept normalization. IEEE Access. 2024;12:147981-90. doi: 10.1109/ACCESS.2024.3472500. | Health information not targeted to laypeople |
| Shaib C, Li ML, Joseph S, Marshall IJ, Li JJ, Wallace BC. Summarizing, simplifying, and synthesizing medical evidence using GPT-3 (with varying success). Proceedings of the conference Association for Computational Linguistics Meeting. 2023;2023:1387-407. doi: https://dx.doi.org/10.18653/v1/2023.acl-short.119. | Health information not targeted to laypeople |
| Waters M. AI meets informed consent: a new era for clinical trial communication. JNCI Cancer Spectrum. 2025;9(2):pkaf028. PMID: 2038201452. doi: https://dx.doi.org/10.1093/jncics/pkaf028. | Ineligible outcomes |
| Bhowmik S, Jamatia A, Rudrapal D, Chakma K, editors. Biomedical lay summarization: Research progress and challenges. 2025 3rd International Conference on Intelligent Systems, Advanced Computing and Communication (ISACC); 2025 27-28 Feb. 2025. IEEE Xplore. | Ineligible outcomes |
| Parillo M, Vaccarino F, Beomonte Zobel B, Mallio CA. ChatGPT and radiology report: potential applications and limitations. La Radiologia medica. 2024;129(12):1849 EP - 63. PMID: 645758609. doi: https://dx.doi.org/10.1007/s11547-024-01915-7. | Ineligible outcomes |
| Amin KS, Forman HP, Davis MA. Even with ChatGPT, race matters. Clinical imaging. 2024;109:110113. doi: https://dx.doi.org/10.1016/j.clinimag.2024.110113. | Ineligible outcomes |
| Oniani D, Sreekumar S, DeAlmeida R, DeAlmeida D, Hui V, Lee YJ, et al. Toward improving health literacy in patient education materials with neural machine translation models. AMIA Joint Summits on Translational Science proceedings AMIA Joint Summits on Translational Science. 2023;2023:418-26. | Ineligible outcomes |
| Phatak A, Savage DW, Ohle R, Smith J, Mago V. Medical text simplification using reinforcement learning (TESLEA): Deep learning-based text simplification approach. JMIR medical informatics. 2022;10(11):e38095. doi: https://dx.doi.org/10.2196/38095. | Ineligible outcomes |
| Ondov B, Attal K, Demner-Fushman D. A survey of automated methods for biomedical text simplification. Journal of the American Medical Informatics Association. 2022;29(11):1976 EP - 88. PMID: 2024425212. doi: https://dx.doi.org/10.1093/jamia/ocac149. | Ineligible outcomes |
| Moramarco F, Juric D, Savkov A, Flann J, Lehl M, Boda K, et al. Towards more patient friendly clinical notes through language models and ontologies. AMIA Annual Symposium proceedings AMIA Symposium. 2021;2021:881 EP - 90. PMID: 637559721. | Ineligible outcomes |
| Sabu S, Baumgartner C, Rajendran P, Turriff L, Mahathanthila NY, Chesover A, et al. GENERATE-THEN-CLASSIFY: CAN LARGE LANGUAGE MODELS GENERATE BETTER SUMMARIES OF RADIOLOGY REPORTS FOR AUTOMATED REPORT CLASSIFICATION? BMJ Paediatrics Open. 2025;9(Supplement 1):A7 EP - A8. PMID: 646475930. doi: https://dx.doi.org/10.1136/bmjpo-2025-GOSH.16. | Ineligible publication type |
| Nguyen AT, Li RA, Gosain AK, Galiano RD. Readability of online patient education materials for cleft care: A systematic review and meta-analysis. Cleft Palate Craniofacial Journal. 2025. PMID: 2033703931. doi: https://dx.doi.org/10.1177/10556656251327803. | Ineligible publication type |
| Nasra M, Jaffri R, Pavlin-Premrl D, Kok HK, Khabaza A, Barras C, et al. Can artificial intelligence improve patient educational material readability? A systematic review and narrative synthesis. Internal medicine journal. 2025;55(1):20-34. doi: https://dx.doi.org/10.1111/imj.16607. | Ineligible publication type |
| Khazanchi R, Chen A, Follett M, Staub J, Hsu W, Patel A, et al. Automatically translating ulmbar spine MRI Reports into patient-facing text: A pilot analysis of GPT-4. Neurosurgery. 2025;71(Supplement 1):191 EP - 2. PMID: 646889895. | Ineligible publication type |
| Chen D, Avison K, Alnassar S, Huang RS, Raman S. Medical accuracy of artificial intelligence chatbots in oncology: a scoping review. Oncologist. 2025;30(4):1-13. doi: 10.1093/oncolo/oyaf038. | Ineligible publication type |
| Asensio-Gomez L, Enriquez BS, Moret RA, Vera BM, Castillo Marcos C, Rubio-Perez I. 39 Can AI explain surgical infection conditions better than surgical residents? A qualitative pilot study. British Journal of Surgery. 2025;112(Supplement 4):iv7. PMID: 646793132. doi: https://dx.doi.org/10.1093/bjs/znaf024.015. | Ineligible publication type |
| Zhang Y, Shi M, Liebman DL, Barna L, Pasquale LR, Elze T, et al. Evaluation of the accuracy of AI-generated clinical summaries from Glaucoma outpatient visits. Investigative Ophthalmology and Visual Science. 2024;65(7):1641. PMID: 645262821. | Ineligible publication type |
| Spinos D, Okonkwo O. Validating artificial intelligence generated patient information leaflets: A patient survey. British Journal of Surgery. 2024;111(Supplement 6):vi3. PMID: 644997370. doi: https://dx.doi.org/10.1093/bjs/znae163.011. | Ineligible publication type |
| Salam B, Kravchenko D, Mesropyan N, Odenthal A, Nowak S, Sprinkart AM, et al. Chatgpt makes cardiovascular MRI reports easy-to-understand: A feasibility study. Journal of Cardiovascular Magnetic Resonance. 2024;26(Supplement 1):100881. PMID: 2031643587. doi: https://dx.doi.org/10.1016/j.jocmr.2024.100881. | Ineligible publication type |
| Rodler S, Ramacciotti LS, Checcucci E, De Backer P, Belenchon IR, Taraktin M, et al. Exploring the efficiency of generative artificial intelligence in rapidly and accurately producing patient information for urological malignancy treatments aligned with the latest EAU guidelines. European Urology. 2024;85(Supplement 1):S947 EP - S8. PMID: 2030740876. doi: https://dx.doi.org/10.1016/S0302-2838%2824%2900773-5. | Ineligible publication type |
| Li H, Moon J, Ricci J, Sim N, Newsome J, Gichoya J. Abstract No. 202 Application of GPT-4 in creating lay person summaries of interventional radiology procedural reports in multiple languages. Journal of Vascular and Interventional Radiology. 2024;35(3 Supplement):S91. PMID: 2030429589. doi: https://dx.doi.org/10.1016/j.jvir.2023.12.241. | Ineligible publication type |
| Ingawale S, Pena O, Chennakesavulu PV, Mishra M. Can artificial intelligence help improve medical literacy in patients with educational disparities? A pilot study assessing the role of Chat GPT in colonoscopy (CS) and esophagogastroduodenoscopy (EGD) patient education. American Journal of Gastroenterology. 2024;119(10 Supplement):S601 EP - S2. PMID: 646034049. doi: https://dx.doi.org/10.14309/01.ajg.0001032844.72900.78. | Ineligible publication type |
| Griffith S, Gwillim E, Yosipovitch G. 52215 The readability of online atopic dermatitis resources compared to ChatGPT's readability and adaptability. Journal of the American Academy of Dermatology. 2024;91(3 Supplement):AB91. PMID: 2034272381. doi: https://dx.doi.org/10.1016/j.jaad.2024.07.369. | Ineligible publication type |
| Gauthier M, Egan S, Johnson N. PCR262 The use of artificial intelligence chat to improve readability of patient-facing materials. Value in Health. 2024;27(6 Supplement):S345. PMID: 2032788938. doi: https://dx.doi.org/10.1016/j.jval.2024.03.2140. | Ineligible publication type |
| Fereydouni P, Spina A, Tang J, Andalib S, Picton B, Fox A. Addressing health-care disparities in patient comprehension using GPT-4. Investigative Ophthalmology and Visual Science. 2024;65(7):357. PMID: 645261246. | Ineligible publication type |
| Dihan Q, Brown AD, Zaldivar A, Chauhan MZ, Eleiwa TK, Hassan AK, et al. Leveraging AI chatbots to transform patient education on idiopathic intracranial hypertension. Investigative Ophthalmology and Visual Science. 2024;65(7):350. PMID: 645260992. | Ineligible publication type |
| Cui D, Li G, Lin M, Mathews P, Akpek EK. Utilization of artificial intelligence to increase usability of cataract surgery patient education websites. Investigative Ophthalmology and Visual Science. 2024;65(7):356. PMID: 645261196. | Ineligible publication type |
| Brown AD, Dihan Q, Chauhan MZ, Eleiwa TK, Hassan AK, Sallam AB, et al. Large language models: A new frontier in pediatric cataract patient education. Investigative Ophthalmology and Visual Science. 2024;65(7):347. PMID: 645260812. | Ineligible publication type |
| Baboun D, Gwillim E, Keri J. 54706 The readability of online rosacea resources compared to ChatGPT's readability and adaptability. Journal of the American Academy of Dermatology. 2024;91(3 Supplement):AB333. PMID: 2034261648. doi: https://dx.doi.org/10.1016/j.jaad.2024.07.1326. | Ineligible publication type |
| Aydin S, Karabacak M, Vlachos V, Margetis K. Large language models in patient education: a scoping review of applications in medicine. Frontiers in Medicine. 2024;11:1477898. PMID: 2032213748. doi: https://dx.doi.org/10.3389/fmed.2024.1477898. | Ineligible publication type |
| Anderer S, Hswen Y. Will generative AI tools improve access to reliable health information? JAMA. 2024;331(16):1347-9. doi: https://dx.doi.org/10.1001/jama.2023.23003. | Ineligible publication type |
| Abdelgadir Y, Thongprayoon C, Craici I, Cheungpasitporn W, Miao J. Enhancing patient comprehension of glomerular disease terminology: Role of Artificial Intelligence (AI) in simplifying medical communication. Journal of the American Society of Nephrology. 2024;35:748. PMID: 645710288. | Ineligible publication type |
| Spina A, Tang J, Picton B, Spiegel S. Using ChatGPT to improve patient accessibility to neuro-ophthalmology research. Journal of the Neurological Sciences. 2023;455(Supplement):122104. PMID: 2029447618. doi: https://dx.doi.org/10.1016/j.jns.2023.122104. | Ineligible publication type |
| McMinn D, Valena T, Bender W. Seeing it in the flesch: Comparing readability between AI-generated and human-written plain-language abstracts. Value in Health. 2023;26(12 Supplement):S11. PMID: 2029284153. doi: https://dx.doi.org/10.1016/j.jval.2023.09.058. | Ineligible publication type |
| Khanmammadova N, Gevorkyan R, Shahait M, Epino M, Nguyen TT, Ali SN, et al. Unlocking patient-centered narratives: Evaluating the feasibility and effectiveness of ChatGPT in generating surgical pathology reports for radical prostatectomy. Journal of Endourology. 2023;37(Supplement 1):A39 EP - A40. PMID: 642827394. doi: https://dx.doi.org/10.1089/end.2023.36001.abstracts. | Ineligible publication type |
| Khanmammadova N, Gevorkyan R, Epino M, Nguyen TT, Ali SN, Cumpanas AD, et al. Revolutionizing prostate biopsy results: Exploring urological surgeons' perspective on ChatGPT-generated patient-centered reports. Journal of Endourology. 2023;37(Supplement 1):A39. PMID: 642827383. doi: https://dx.doi.org/10.1089/end.2023.36001.abstracts. | Ineligible publication type |
| Collins L, Pinero LM, Keenan E, Finkel D. Using AI-generated patient information sheets on colonoscopies to improve doctor-patient communication. American Journal of Gastroenterology. 2023;118(10 Supplement):S1302. PMID: 646961019. | Ineligible publication type |
| Atmar A, Andalib S, Spina A, Dylewski J, Haspil-Corgan T. ChatGPT has the potential to increase accessibility to cardiology research. Circulation. 2023;148(Supplement 1). PMID: 642972103. doi: https://dx.doi.org/10.1161/circ.148.suppl_1.18238. | Ineligible publication type |
| Amin K, Khosla P, Doshi R, Chheang S, Forman HP. Artificial intelligence to improve patient understanding of radiology reports. Yale Journal of Biology and Medicine. 2023;96(3):407 EP - 14. PMID: 2026160425. doi: https://dx.doi.org/10.59249/NKOY5498. | Ineligible publication type |
| Tripathi S, Dako F. The potential of large language models for radiology report simplification and translations. Journal of the American College of Radiology. 2024;21(12):1896 EP - 7. PMID: 2033639625. doi: https://dx.doi.org/10.1016/j.jacr.2024.06.004. | No peer-review publication |
| Malkani K, Zhang R, Falk Z, Tawde P, Hughes R, Parker M, et al. Simplifying cardiology research abstracts: Assessing ChatGPT's readability and comprehensibility for non- medical audiences. Circulation. 2024;150(Supplement 1). PMID: 646742361. doi: https://dx.doi.org/10.1161/circ.150.suppl_1.4116844. | No peer-review publication |
| King R, Samaan J, Ghashghaei R, Bharani V, Haquang J. ChatGPT-4 improves readability of institutional heart failure patient education materials. Circulation. 2024;150(Supplement 1). PMID: 646742473. doi: https://dx.doi.org/10.1161/circ.150.suppl_1.4145543. | No peer-review publication |
| Yang X, Xiao Y, Zhang Y, Deng H, Huang J, Shi H, et al. Enhancing doctor-patient communication using large language models for pathology report interpretation. BMC medical informatics and decision making. 2025;25(1):36. PMID: 646395173. doi: https://dx.doi.org/10.1186/s12911-024-02838-z. | No patient education material |
| Yang X, Xiao Y, Shi H, Deng H, Huang J, Zhang Y, et al. Enhancing physician-patient communication in oncology using GPT-4 through simplified radiology reports: Multicenter quantitative study. Journal of Medical Internet Research. 2025;27:e63786. PMID: 2038384836. doi: https://dx.doi.org/10.2196/63786. | No patient education material |
| Rinderknecht E, Schmelzer A, Kravchuk A, Gosler C, Breyer J, Gilfrich C, et al. Leveraging large language models for high-quality lay summaries: Efficacy of ChatGPT-4 with custom prompts in a consecutive series of prostate cancer manuscripts. Current oncology (Toronto, Ont). 2025;32(2). doi: https://dx.doi.org/10.3390/curroncol32020102. | No patient education material |
| Pavicic JS, Marusic A, Buljan I. Using ChatGPT to improve the presentation of plain language summaries of Cochrane Systematic Reviews About oncology interventions: Cross-sectional Study. JMIR Cancer. 2025;11:e63347. PMID: 2038080562. doi: https://dx.doi.org/10.2196/63347. | No patient education material |
| Li HH, Moon JT, Kumar S, Ricci J, Sim N, Bercu ZL, et al. Evaluation of multilingual simplifications of IR procedural reports using GPT-4. Journal of Vascular and Interventional Radiology. 2025;36(4):696 EP - 703.e1. PMID: 2037511484. doi: https://dx.doi.org/10.1016/j.jvir.2025.01.002. | No patient education material |
| Gupta A, Singh S, Malhotra H, Pruthi H, Sharma A, Garg AK, et al. Provision of radiology reports simplified with large language models to patients with cancer: Impact on patient satisfaction. JCO Clinical Cancer Informatics. 2025;9:e2400166. PMID: 2037338799. doi: https://dx.doi.org/10.1200/CCI-24-00166. | No patient education material |
| Gupta A, Rastogi A, Malhotra H, Rangarajan K. Comparative evaluation of large language models for translating radiology reports into Hindi. Indian Journal of Radiology &amp; Imaging. 2025;35(1):88-96. doi: 10.1055/s-0044-1789618. | No patient education material |
| Guerra GA, Grove S, Le J, Hofmann HL, Shah I, Bhagavatula S, et al. Artificial intelligence as a modality to enhance the readability of neurosurgical literature for patients. Journal of Neurosurgery. 2025;142(4):1189 EP - 95. PMID: 2038148229. doi: https://dx.doi.org/10.3171/2024.6.JNS24617. | No patient education material |
| Eisinger F, Holderried F, Mahling M, Stegemann-Philipps C, Herrmann-Werner A, Nazarenus E, et al. What's going on with me and how can I better manage my health? The potential of GPT-4 to transform discharge letters into patient-centered letters to enhance patient safety: Prospective, exploratory study. Journal of Medical Internet Research. 2025;27:e67143. PMID: 2037120834. doi: https://dx.doi.org/10.2196/67143. | No patient education material |
| Cosma C, Radi A, Cattano R, Zanobini P, Bonaccorsi G, Lorini C, et al. Potential role of ChatGPT in simplifying and improving informed consent forms for vaccination: a pilot study conducted in Italy. BMJ health & care informatics. 2025;32(1). doi: https://dx.doi.org/10.1136/bmjhci-2024-101248. | No patient education material |
| Cork S, Hopcroft K. Evaluating ChatGPT for converting clinic letters into patient-friendly language. BJGP open. 2025. doi: https://dx.doi.org/10.3399/BJGPO.2024.0300. | No patient education material |
| Can E, Uller W, Vogt K, Doppler MC, Busch F, Bayerl N, et al. Large language models for simplified interventional radiology reports: A comparative analysis. Academic Radiology. 2025;32(2):888 EP - 98. PMID: 2034839928. doi: https://dx.doi.org/10.1016/j.acra.2024.09.041. | No patient education material |
| Cadiente A, Implicito C, Udaiyar A, Ho A, Wan C, Chen J, et al. Evaluating incontinence abstracts: Artificial intelligence-generated versus Cochrane Review. Urogynecology. 2025. PMID: 2038526909. doi: https://dx.doi.org/10.1097/SPV.0000000000001688. | No patient education material |
| Zaretsky J, Kim JM, Baskharoun S, Zhao Y, Austrian J, Aphinyanaphongs Y, et al. Generative artificial intelligence to transform inpatient discharge summaries to patient-friendly language and format. JAMA Network Open. 2024;7(3):e240357. PMID: 2030917280. doi: https://dx.doi.org/10.1001/jamanetworkopen.2024.0357. | No patient education material |
| Tepe M, Emekli E. Decoding medical jargon: The use of AI language models (ChatGPT-4, BARD, microsoft copilot) in radiology reports. Patient Education and Counseling. 2024;126:108307. PMID: 2032203725. doi: https://dx.doi.org/10.1016/j.pec.2024.108307. | No patient education material |
| Steimetz E, Minkowitz J, Gabutan EC, Ngichabe J, Attia H, Hershkop M, et al. Use of artificial intelligence chatbots in interpretation of pathology reports. JAMA Network Open. 2024;7(5):E2412767. PMID: 2032483079. doi: https://dx.doi.org/10.1001/jamanetworkopen.2024.12767. | No patient education material |
| Sridharan K, Sivaramakrishnan G. Enhancing readability of USFDA patient communications through large language models: a proof-of-concept study. Expert review of clinical pharmacology. 2024;17(8):731-41. doi: https://dx.doi.org/10.1080/17512433.2024.2363840. | No patient education material |
| Salam B, Kravchenko D, Nowak S, Sprinkart AM, Weinhold L, Odenthal A, et al. Generative pre-trained transformer 4 makes cardiovascular magnetic resonance reports easy to understand. Journal of Cardiovascular Magnetic Resonance. 2024;26(1):101035. PMID: 2031171051. doi: https://dx.doi.org/10.1016/j.jocmr.2024.101035. | No patient education material |
| Moons P, Van Bulck L. Using ChatGPT and Google Bard to improve the readability of written patient information: a proof of concept. European journal of cardiovascular nursing. 2024;23(2):122-6. doi: https://dx.doi.org/10.1093/eurjcn/zvad087. | No patient education material |
| Maroncelli R, Rizzo V, Pasculli M, Cicciarelli F, Macera M, Galati F, et al. Probing clarity: AI-generated simplified breast imaging reports for enhanced patient comprehension powered by ChatGPT-4o. European Radiology Experimental. 2024;8(1):124. PMID: 2032072827. doi: https://dx.doi.org/10.1186/s41747-024-00526-1. | No patient education material |
| Leroy G, Kauchak D, Harber P, Pal A, Shukla A. Text and audio simplification: Human vs. ChatGPT. AMIA Joint Summits on Translational Science proceedings AMIA Joint Summits on Translational Science. 2024;2024:295-304. | No patient education material |
| Kuckelman IJ, Wetley K, Yi PH, Ross AB. Translating musculoskeletal radiology reports into patient-friendly summaries using ChatGPT-4. Skeletal Radiology. 2024;53(8):1621 EP - 4. PMID: 2028065159. doi: https://dx.doi.org/10.1007/s00256-024-04599-2. | No patient education material |
| Kim H, Jin HM, Jung YB, You SC. Patient-friendly discharge summaries in Korea based on ChatGPT: Software development and validation. Journal of Korean medical science. 2024;39(16):e148. PMID: 644139396. doi: https://dx.doi.org/10.3346/jkms.2024.39.e148. | No patient education material |
| Kianian R, Sun D, Rojas-Carabali W, Agrawal R, Tsui E. Large language models may help patients understand peer-reviewed scientific articles about ophthalmology: Development and usability study. Journal of Medical Internet Research. 2024;26:e59843. PMID: 2036666843. doi: https://dx.doi.org/10.2196/59843. | No patient education material |
| Jeblick K, Schachtner B, Dexl J, Mittermeier A, Stuber AT, Topalis J, et al. ChatGPT makes medicine easy to swallow: an exploratory case study on simplified radiology reports. European Radiology. 2024;34(5):2817 EP - 25. PMID: 2025897958. doi: https://dx.doi.org/10.1007/s00330-023-10213-1. | No patient education material |
| Güneş YC, Cesur T, Çamur E. Comparative analysis of large language models in simplifying Turkish ultrasound reports to enhance patient understanding. European Journal of Therapeutics. 2024;30(5):714-23. doi: 10.58600/eurjther2225. | No patient education material |
| Gulati V, Roy SG, Moawad A, Garcia D, Babu A, Poot JD, et al. Transcending language barriers: Can ChatGPT be the key to enhancing multilingual accessibility in health care? Journal of the American College of Radiology : JACR. 2024;21(12):1888-95. doi: https://dx.doi.org/10.1016/j.jacr.2024.05.009. | No patient education material |
| Gill B, Bonamer J, Kuechly H, Gupta R, Emmert S, Kurkowski S, et al. ChatGPT is a promising tool to increase readability of orthopedic research consents. Journal of Orthopaedics, Trauma and Rehabilitation. 2024;31(2):148 EP - 52. PMID: 2028039929. doi: https://dx.doi.org/10.1177/22104917231208212. | No patient education material |
| Doshi R, Amin KS, Khosla P, Bajaj SS, Chheang S, Forman HP. Quantitative evaluation of large language models to streamline radiology report impressions: A multimodal retrospective analysis. Radiology. 2024;310(3):e231593. doi: https://dx.doi.org/10.1148/radiol.231593. | No patient education material |
| Dehkordi MKH, Zhou S, Perl Y, Deek FP, Einstein AJ, Elhanan G, et al., editors. Enhancing patient comprehension: An effective sequential prompting approach to simplifying EHRs using LLMs. 2024 IEEE International Conference on Bioinformatics and Biomedicine (BIBM); 2024 3-6 Dec. 2024. IEEE Xplore. | No patient education material |
| Butler JJ, Puleo J, Harrington MC, Dahmen J, Rosenbaum AJ, Kerkhoffs GMMJ, et al. From technical to understandable: Artificial Intelligence Large Language Models improve the readability of knee radiology reports. Knee surgery, sports traumatology, arthroscopy : official journal of the ESSKA. 2024;32(5):1077 EP - 86. PMID: 643763667. doi: https://dx.doi.org/10.1002/ksa.12133. | No patient education material |
| Butler JJ, Harrington MC, Tong Y, Rosenbaum AJ, Samsonov AP, Walls RJ, et al. From jargon to clarity: Improving the readability of foot and ankle radiology reports with an artificial intelligence large language model. Foot and ankle surgery : official journal of the European Society of Foot and Ankle Surgeons. 2024;30(4):331-7. doi: https://dx.doi.org/10.1016/j.fas.2024.01.008. | No patient education material |
| Sarangi PK, Lumbani A, Swarup MS, Panda S, Sahoo SS, Hui P, et al. Assessing ChatGPT's proficiency in simplifying radiological reports for healthcare professionals and patients. Cureus. 2023;15(12):e50881. doi: https://dx.doi.org/10.7759/cureus.50881. | No patient education material |
| Lyu Q, Tan J, Zapadka ME, Ponnatapura J, Niu C, Myers KJ, et al. Translating radiology reports into plain language using ChatGPT and GPT-4 with prompt learning: results, limitations, and potential. Visual computing for industry, biomedicine, and art. 2023;6(1):9. doi: https://dx.doi.org/10.1186/s42492-023-00136-5. | No patient education material |
| Li H, Moon JT, Iyer D, Balthazar P, Krupinski EA, Bercu ZL, et al. Decoding radiology reports: Potential application of OpenAI ChatGPT to enhance patient understanding of diagnostic reports. Clinical imaging. 2023;101:137-41. doi: https://dx.doi.org/10.1016/j.clinimag.2023.06.008. | No patient education material |
| Eppler MB, Ganjavi C, Knudsen JE, Davis RJ, Ayo-Ajibola O, Desai A, et al. Bridging the gap between urological research and patient understanding: The role of large language models in automated generation of layperson's summaries. Urology Practice. 2023;10(5):436 EP - 43. PMID: 2026930007. doi: https://dx.doi.org/10.1097/UPJ.0000000000000428. | No patient education material |
| Chung EM, Zhang SC, Nguyen AT, Atkins KM, Kamrava M. Feasibility and acceptability of ChatGPT generated radiology report summaries for cancer patients. International Journal of Radiation Oncology Biology Physics. 2023;117(2 Supplement):e463. PMID: 2026576462. doi: https://dx.doi.org/10.1016/j.ijrobp.2023.06.1662. | No patient education material |
| Bala S, Keniston A, Burden M. Patient perception of plain-language medical notes generated using artificial intelligence software: Pilot mixed-methods study. JMIR formative research. 2020;4(6):e16670. doi: https://dx.doi.org/10.2196/16670. | No patient education material |
