# Supplementary figures and images for: Automated Approaches of Text Simplification of Patient Education Materials: Scoping Review
Source: J Med Internet Res. 2026 May 7;28:e88365. doi: 10.2196/88365 (PMC13195379; doi:10.2196/88365)

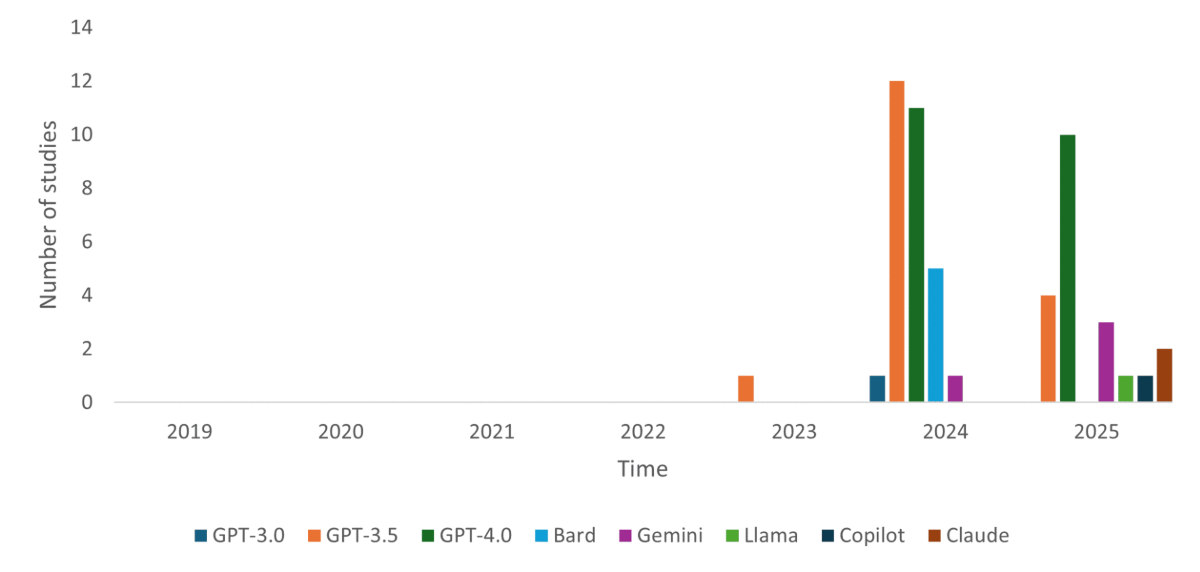

Supplement: Multimedia Appendix 5 [file jmir_v28i1e88365_app5.png]
