# Supplementary material for: Automated Approaches of Text Simplification of Patient Education Materials: Scoping Review
Source: J Med Internet Res. 2026 May 7;28:e88365. doi: 10.2196/88365 (PMC13195379; doi:10.2196/88365)
Supplement: Multimedia Appendix 6 [file jmir_v28i1e88365_app6.docx]

**Multimedia Appendix 5 – Overview of prompts used for automatic text simplification in the included studies.**

| Author, Year | LLM | Prompt |
| --- | --- | --- |
| Spina 2025 [38] | GPT-4.0 | Please rewrite this text to be readable at a 5th grade level. Do not include information not contained in the original text, and do not exclude information contained in the original text. |
| Reaver 2025 [39] | GPT-4.0 | EN: Rewrite this document at a sixth-grade reading level focusing on reducing the use of medical jargon, reducing the number of syllables per word, and shortening sentence length. Use the following readability formulas as your criteria: Fry Readability Score, Flesch-Reading Ease Index, SMOG Index, LIX Readability Index, and RIX Readability Index. |
|  |  | ES: Rewrite this document at a sixth-grade reading level in Spanish focusing on reducing the use of medical jargon, reducing the number of syllables per word, and shortening sentence length. Use the following readability formulas as your criteria: Gilliam Peña Mountain Fry Graph, Fernandez-Huerta Readability Index, SMOG SOL Index, LIX Readability Index, and RIX Readability Index. |
| Picton 2025 [40] | GPT-4.0 | Please rewrite the following text to be readable at a 5th-grade level. Do not include information not contained in the original text, and do not exclude information contained within the original text. |
| Li 2025 [48] | GPT 3.5 | Simplify to a fifth-grade reading level. |
| Dihan 2025 [57] | GPT 3.5 | Given patient education materials are recommended to be written at a 6th grade reading level, using the SMOG readability formula, can you rewrite the following text to a 6th grade reading level: [insert text]. |
|  | GPT-4.0 |  |
|  | Gemini Advanced |  |
| Chandra 2025 [41] | GPT-4.0 | Rewrite this text at a sixth-grade to eighth grade level without losing information. |
| Busigo Torres 2025 [42] | GPT-4.0 | nr |
| Andalib 2025 [66] | GPT-3.5 | Please rewrite this text to be readable at a fifth-grade level. Do not include information not contained in the original text, and do not exclude information in the original text. |
|  | GPT-4.0 |  |
|  | Claude 2 |  |
|  | Llama 2 |  |
| Will 2025 [58] | GPT-4.0 | Translate to a fifth-grade reading level. |
|  | Gemini 1.5-flash |  |
|  | Claude 3.5 Sonnet |  |
| Naghdi 2025 [59] | GPT-3.5 | Rewrite this text for individuals with low literacy. |
|  | GPT-4.0 |  |
|  | Copilot |  |
|  | Gemini |  |
| Singh 2025 [43] | GPT-4.0 | Revise the following text to align with an 8th-grade reading level (ages 13-14), adhering to these strict criteria:  Flesch Reading Ease Score Above 60:  Sentence Length: Limit each sentence to a maximum of 20 words.  Word Syllables: Select words that have, on average, no more than 1.5 syllables.  Flesch-Kincaid Grade Level Between 7 and 8:  Sentence Structure: Restrict sentences to a brief, clear structure, maintaining an average length below 20 words.  Syllable Count: Opt for words with fewer syllables to reduce the overall grade level.  SMOG Index No Higher Than 8:  Polysyllabic Words: Limit polysyllabic words (three or more syllables) to less than 5% of the total word count.  Sentence Complexity: Avoid complex sentence constructions. Prioritize straightforward and direct sentences.  Paragraph and Sentence Construction:  Paragraph Length: Each paragraph should contain no more than 4-5 sentences.  Voice: Use the active voice predominantly. Minimize passive voice constructions.  Sentence Complexity: Avoid complex or compound sentences. Focus on simple sentence structures.  The content topic is [disease/pathology]. The revised text must accurately and clearly convey key concepts/information related to this topic. The goal is to ensure the text is easily understandable, maintains its informative integrity, and strictly adheres to the readability standards outlined above. |
| Zaki 2024 [44] | GPT-4.0 | Convert the following to a fifth-grade reading level. |
| Vallurupalli 2024 [49] | GPT 3.5 | Rewrite this paragraph at a 6th to 8th grade level without losing information from the original paragraph. |
| Shehab 2024 [45] | GPT-4.0 | nr |
| Patel 2024 [46] | GPT-4.0 | Translate this text to a sixth-grade reading level. |
| Oliva 2024 [50] | GPT 3.5 | Translate to a 5th-grade reading level. |
| Kianian 2024 [61] | GPT 4.0 | Considering that the average American reads at a 6th grade level, using the FKGL formula, can you rewrite the following text to 6th grade level: [insert text]. |
|  | Google Bard |  |
| Gupta 2024 [51] | GPT 3.5 | EN: Can you make this text easier to read? |
|  |  | ES: ¿Puedes hacer esto más fácil de leer? |
| Gupta 2024 [60] | GPT 4.0 | Please, reformulate this text to a sixth-grade reading level. |
|  | Gemini |  |
| Garcia Valencia 2024 [67] | GPT 3.5 | Please modify the text to make it easier to understand for someone who reads at or below an 8th grade level. |
|  | GPT-4.0 |  |
| Fanning 2024 [68] | GPT 3.5 | General prompt: Re-write the following transcript at a 6th-grade level. Use clear and simple language, even when explaining complex topics. Bias toward short sentences. Avoid jargon and acronyms. Maintain the general structure of titles and subtitles. Specific prompt: Please enhance the readability of the provided text for a sixth-grade audience while ensuring there is no change in meaning or information. Aim for a score of approximately 6.0 on the Flesch-Kincaid Grade Level, Gunning Fog Index, Fry, Raygor Estimate, and SMOG readability scales. You can simplify sentence structures, replace complex words with simpler alternatives, and make necessary changes to achieve the desired readability level. Your goal is to make the text more accessible and comprehensible for a typical sixth-grade student without altering the original message and content. |
|  | GPT-4.0 |  |
| Dihan 2024 [62] | GPT-3.5 | Given patient education materials are recommended to be written at a 6th-grade reading level, using the SMOG readability formula, can you rewrite the following text to a 6th- grade reading level: [insert text]. |
|  | GPT-4.0 |  |
|  | Google Bard |  |
| Dihan 2024 [63] | GPT-3.5 | Given the average American reads at a 6th-grade reading level, using the SMOG readability formula, can you rewrite the following text to a 6th-grade reading level: [insert text] |
|  | GPT-4.0 |  |
|  | Google Bard |  |
| Dihan 2024 [64] | GPT-3.5 | Given PEMs are recommended to be written at a sixth-grade reading level, using the SMOG readability formula, can you rewrite the following text to a sixth-grade reading level: (insert text) |
|  | GPT-4.0 |  |
|  | Google Bard |  |
| Baldwin 2024 [56] | GPT-3.0 | Revise the provided patient education material to have a readability level comprehensible by an individual with the literacy level of an 11-year-old, while maintaining a tone suitable for an adult audience. |
| Ayre 2024 [52] | GPT-3.5 | Rewrite the text for people with low literacy. |
| Manasyan 2024 [53] | GPT-3.5 | Rewrite at a 5th Flesch-Kincaid Grade Level. |
| Vallurupalli 2024 [54] | GPT-3.5 | Rewrite this paragraph for an 8th grader without losing information from the original paragraph. |
| Abreu 2024 [47] | GPT-4.0 | Using the Flesch-Kincaid Grade Level, Fry Readability Score, Gunning Fog Score, and the SMOG Index formulas as your criteria. Rewrite the following text aiming for a 6th-grade readability level. |
| Rouhi 2024 [65] | GPT-3.5 | Translate to 5th-grade reading level. |
|  | Google Bard |  |
| Kirchner 2023 [55] | GPT-3.5 | Translate to fifth-grade reading level. |

EN = English; ES = Spanish, nr = not reported
