# Supplementary material for: Automated Approaches of Text Simplification of Patient Education Materials: Scoping Review
Source: J Med Internet Res. 2026 May 7;28:e88365. doi: 10.2196/88365 (PMC13195379; doi:10.2196/88365)
Supplement: Multimedia Appendix 7 [file jmir_v28i1e88365_app7.docx]

**Multimedia Appendix 6 - Overview of reported outcomes**

| Author | Linguistic quality | | | | | | | | | | Content fidelity | | | | Overall understandability^e^ |
| --- | --- | --- | --- | --- | --- | --- | --- | --- | --- | --- | --- | --- | --- | --- | --- |
|  | Linguistic comprehensibility | | | | | | | | | | Factual correctness | | Factual completeness | |  |
|  | Readability formulas | | Text length | | Lexical complexity | | Syntactic complexity | | Vocabulary complexity | |  | |  | |  |
|  | Readability metric | Reading grade level | Word count | Sentence count | Word length | Syllables^a^ | Passive voice | Sentence length | Acronyms, uncommon words, medical jargon | Hard words^c^ | Content similarity | Content accuracy | Content retention | Content omission^d^ |  |
| Spina 2025 [21] | A | A |  |  |  |  |  |  |  |  | A |  |  |  |  |
| Reaver 2025 [22] | A | A | A |  |  | A |  | A |  |  | A | A |  |  |  |
| Picton 2025 [23] | A | A | A |  |  |  |  |  |  |  | A+H | A+H |  |  |  |
| Li 2025 [24] | A | A |  |  |  |  |  |  |  |  |  |  |  |  |  |
| Dihan 2025 [25] | A | A | A | A |  | A |  |  |  |  |  | H |  |  | H^f^ |
| Dihan 2024 [42] | A | A | A | A |  | A |  |  |  |  |  | H |  |  | H^f^ |
| Dihan 2024 [43] | A | A | A | A |  | A |  |  |  |  |  | H |  |  | H^f^ |
| Dihan 2024 [44] | A | A | A |  |  | A |  |  |  |  |  |  |  |  |  |
| Chandra 2025 [26] | A | A |  |  |  |  |  |  |  |  |  |  |  |  |  |
| Busigo Torres 2025 [27] | A | A | A |  |  |  |  |  |  |  |  | H |  |  |  |
| Andalib 2025 [28] | A | A | A | A |  | A |  |  |  |  | A+H |  |  |  |  |
| Zaki 2024 [32] | A | A |  |  |  |  |  |  |  |  |  |  |  |  |  |
| Vallurupalli 2024 [33] | A | A |  |  |  |  |  |  |  |  |  |  |  |  |  |
| Shehab 2024 [34] | A | A | A |  |  |  |  |  |  |  |  |  |  |  |  |
| Patel 2024 [35] | A | A |  |  |  |  |  |  |  |  |  | H | H |  |  |
| Oliva 2024 [36] | A | A |  |  |  |  |  |  |  |  |  | H | H |  |  |
| Kianian 2024 [37] | A | A |  |  |  |  |  |  |  |  |  |  |  |  |  |
| Sudharshan 2024 [38] | A |  |  |  |  |  |  |  |  |  |  |  |  |  |  |
| Gupta 2024 [39] | A | A | A |  |  |  |  |  |  |  |  |  | H |  | H^g^ |
| Garcia Valencia 2024 [40] | A | A |  |  |  |  |  |  |  |  | A | H |  |  | H^h^ |
| Fanning 2024 [41] | A | A |  |  |  |  |  |  |  |  |  |  |  |  |  |
| Baldwin 2024 [45] | A | A |  |  |  |  |  |  |  |  |  |  |  |  |  |
| Ayre 2024 [46] | A | A | A |  |  |  | A |  | H^b^ |  |  |  | H |  |  |
| Kirchner 2023 [51] | A | A |  |  |  |  |  |  |  |  |  | H | H |  |  |
| Manasyan 2024 [47] | A | A | A |  |  | A |  |  |  |  |  |  |  |  |  |
| Vallurupalli 2024 [48] | A | A |  |  |  |  |  |  |  |  |  | H |  |  |  |
| Will 2025 [29] | A | A | A |  |  |  |  |  |  |  |  | H |  |  | H^e^ |
| Naghdi 2025 [30] | A | A | A | A |  |  | A | A |  | A |  | H | H | H |  |
| Singh 2025 [31] | A | A |  |  |  |  |  |  |  |  |  | H |  |  |  |
| Abreu 2024 [49] | A | A |  |  | A | A |  | A |  |  | A | A |  |  |  |
| Rouhi 2024 [50] | A | A |  |  |  |  |  |  |  |  |  | H |  |  |  |
| Total | n=31 | n=30 | n=14 | n=5 | n=1 | n=8 | n=2 | n=3 | n=1 | n=1 | n=6 | n=16 | n=6 | n=1 | n=6 |

A = automated rating, A+H = both automated and human rating, H = human rating

^a^ as defined in the studies: syllables count, ≥3 syllables, syllables/sentence, and syllables/word, ^b^ combined endpoint, ^c^ hard words: no definition available by the authors of the study, ^d^ excluded details were unnecessary, ^e^ understandability is the degree to which patients from diverse background can process and explain the key messages in a PEM [42], ^f^ measured with the Patient Education Materials Assessment Tool for Printable Materials (PEMAT-U) scale, ^g^ measurement of clarity defined as “The material is clear to the average patient,” ^h^ measurement of clarity and comprehensibility defined as “Assessing whether the modifications indeed made the information more accessible to individuals with lower literacy levels, without sacrificing the depth of information.”
